# Chronic disturbance modulates symbiont (Symbiodiniaceae) beta diversity on a coral reef

**DOI:** 10.1038/s41598-020-60929-z

**Published:** 2020-03-11

**Authors:** Danielle C. Claar, Kristina L. Tietjen, Kieran D. Cox, Ruth D. Gates, Julia K. Baum

**Affiliations:** 1 Department of Biology, University of Victoria, Victoria, British Columbia V8W 2Y2 Canada; 2 Hakai Institute, Calvert Island, British Columbia, Canada; 3 Hawaii Institute of Marine Biology, Kaneohe, HI 96744 USA; 4 University of Washington, School of Aquatic and Fisheries Science, 1122 NE Boat St, Seattle, WA 98105 USA

**Keywords:** Community ecology, Microbial ecology

## Abstract

Chronic disturbance can disrupt ecological interactions including the foundational symbiosis between reef-building corals and the dinoflagellate family Symbiodiniaceae. Symbiodiniaceae are photosynthetic endosymbionts necessary for coral survival, but many Symbiodiniaceae can also be found free-living in the environment. Since most coral species acquire new Symbiodiniaceae from the environment each generation, free-living Symbiodiniaceae represent important pools for coral symbiont acquisition. Yet, little is known about the diversity of, or impacts of disturbance on, free-living Symbiodiniaceae. To determine how chronic and pulse disturbances influence Symbiodiniaceae communities, we sampled three reef habitat compartments - sediment, water, and coral (*Pocillopora grandis, Montipora aequituberculata, Porites lobata*) - at sites exposed to different levels of chronic anthropogenic disturbance, before, during, and after a major storm. Almost no (4%) Symbiodiniaceae amplicon sequence variants (ASVs) were found in all three compartments, and over half were found uniquely in coral. Sites experiencing chronic disturbance were typically associated with higher symbiont beta diversity (i.e., variability and turnover) across reef habitat compartments. Pulse stress, from the storm, exhibited some influence on symbiont beta diversity but the effect was inconsistent. This suggests that in this ecosystem, the effects of chronic disturbance are more prominent than temporal variability during a pulse disturbance for shaping symbiont communities.

## Introduction

Disturbances, both natural and anthropogenic, can alter species diversity and cause stress on ecosystem structure and function. Disturbance can manifest as chronic disturbance (i.e., long-term or press disturbance) or pulse disturbance (i.e., short-term or acute disturbances events)^1,2^. Both types of disturbance can alter not only alpha diversity (i.e., species diversity within a community; e.g.^3,4^) but also beta diversity (i.e., differences in species composition among communities^5,6^), which has emerged as a sensitive indicator of ecosystem change under stress^7,8^. Beta diversity metrics can be split into two working definitions: ‘turnover’ is defined as compositional changes of species assemblages across a gradient, while ‘variation’ is defined as the amount of variability among species assemblages within each community^9^. When communities are analyzed in a multivariate context, ‘turnover’ represents multivariate location, while ‘variation’ represents multivariate spread (beta dispersion). A growing body of literature has shown that for macro-organisms, disturbances often cause a decrease in beta diversity, leading to ‘biotic homogenization’ (i.e., communities become simplified and more similar to one another^10–13^). In micro-organisms, however, emerging evidence suggests that disturbance can have the opposite effect, resulting in elevated beta diversity (e.g.^14–16^; reviewed in^17^). This effect has been attributed to the ‘Anna Karenina’ principle, wherein stress causes an increase in stochastic changes (i.e., variation) and community dispersion within a microbiome, as opposed to directional changes (i.e., turnover) in community structure^17–19^.

Understanding how disturbances alter the diversity of coral reefs is of high priority, given the biological and socioeconomic importance of these diverse tropical ecosystems^20^. One of the most critical and susceptible components of these ecosystems is the symbiosis between scleractinian corals and the diverse dinoflagellate Family Symbiodiniaceae^21^. These coral-algal symbioses are an important component of coral health, but sensitive to chronic stressors, such as poor water quality^22–24^, and pulse disturbances, most notably temperature anomalies^25^. There is significant functional diversity within the Family Symbiodiniaceae^26,27^, and local conditions, including natural environmental variability and chronic human disturbance, act as filters to limit which Symbiodiniaceae are available for coral symbioses^28^. Local conditions are thus important drivers that set the baseline and potential scope of corals’ ability to respond to pulse stress events. Thermal gradients have a strong influence on Symbiodiniaceae biogeography, but chronic local stressors may also influence these patterns^29,30^. When the symbiosis between corals and Symbiodiniaceae breaks down due to environmental stress, corals bleach^25^. Bleaching events are now occurring globally at an alarming rate, exacerbated by global warming and chronic local stressors such as fishing, pollution, and eutrophication^31–33^.

Approximately 80–85% of all scleractinian (reef building) coral species must take up symbionts anew from the environment each generation^34,35^. Therefore, free-living Symbiodiniaceae are vital for the initiation of new symbioses with coral larvae and newly settled recruits, although the timing of acquisition is under selection, and early acquisition in the larval phase may be selected against since symbionts may not provide the same nutritional benefits as they do in older individuals^36^. Symbiodiniaceae in the environment may also provide a source for adult corals to take up new symbionts after disturbance and bleaching^37,38^, but the frequency and ecological relevance of this uptake is contested^39,40^. Symbiodiniaceae found in the environment (“free-living Symbiodiniaceae”) occur in several different reef habitat “compartments”, including sediment, the water column, and as epiphytes on macroalgae and seagrass^41–44^. These free-living Symbiodiniaceae can be divided into two categories: (1) “exclusively free-living Symbiodiniaceae” are those that do not participate in symbioses with eukaryotic hosts^45^; (2) “transiently free-living Symbiodiniaceae” are typically associated with invertebrate hosts but can be transient in the environment^46,47^. It is likely that transiently free-living Symbiodiniaceae do not persist perpetually outside host corals, but rather that these populations are ephemeral and consistently replenished from host sources^48^. The diversity and stability of free-living Symbiodiniaceae communities are therefore likely subject to environmental fluctuations^49,50^.

Although free-living Symbiodiniaceae provide a pool of potentially viable symbionts for coral uptake, little is known about how these Symbiodiniaceae communities vary in response to chronic stressors (which often vary across locations) and pulse stressors (which typically are of short-duration and hence require measurement over time). Initial research showed that free-living Symbiodiniaceae abundance can be spatially variable at local scales, with cells preferentially aggregating in reef habitats^28,42^. Although free-living Symbiodiniaceae communities may vary over space, they can remain consistent over time (~17 months^51^), and thermal variability across habitats may drive differences in Symbiodiniaceae beta diversity^52^. Nonetheless, significant questions remain about the variability of Symbiodiniaceae communities across distinct reef habitat compartments, and how chronic and pulse disturbances influence them, including: (1) Does chronic disturbance change the beta diversity of Symbiodiniaceae communities? (2) Can physical pulse disturbance events instigate ecologically meaningful changes in free-living Symbiodiniaceae beta diversity, thereby altering the availability of certain symbionts for uptake into symbiosis? And (3) Are the impacts of chronic and pulse disturbances consistent across endosymbiotic Symbiodiniaceae in coral species, and free-living Symbiodiniaceae in other reef habitat compartments?

**Figure 1:**
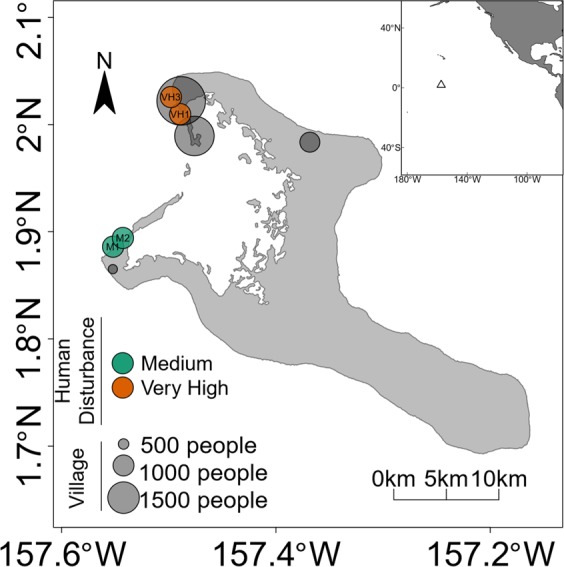
Sample sites on Kiritimati, showing locations with medium (M1 and M2) and high (VH1 and VH3) local human disturbance. Inset shows Kiritimati’s location in the central equatorial Pacific Ocean (open triangle).

In this study, we investigated how chronic and pulse disturbances alter the beta diversity of Symbiodiniaceae communities. First, we examined the influence of chronic local anthropogenic disturbance (fishing pressure, pier infrastructure) by sampling free-living symbionts in the water and sediment, and endosymbiotic symbionts in three dominant coral species at two sites with different intensities of chronic disturbance on Kiritimati Island (Fig. 1; Supplementary Fig. S1). We sequenced the internal transcribed spacer region 2 (ITS2) region of Symbiodiniaceae to determine symbiont sequence assemblages in each sample. We filtered, processed, and analyzed sequences using the dada2 pipeline and used the resulting amplicon sequence variants (ASVs; quality-controlled exact sequence variants^53^) to analyze Symbiodiniaceae sequence assemblages. We hypothesized that, unlike for macro-organisms, chronic disturbance would increase observed Symbiodiniaceae beta diversity in both coral-associated and free-living symbiont communities, causing changes in community structure (i.e., turnover) and increased variation. Second, we tested whether and how a natural acute pulse disturbance, namely a major winter swell event, altered Symbiodiniaceae communities across reef habitat compartments. This swell event was caused by storms from the western Pacific, and it produced waves in the 94^th^ percentile of significant wave height over the previous twelve years (Supplementary Fig. S2). Storm wave events can lead to mechanical homogenization of the water column and suspension of the benthos, as well as releasing Symbiodiniaceae from corals through coral injury and death and may provide important opportunities for exchange among these reef habitat compartments. We hypothesized that physical disturbance caused by this major storm swell event would homogenize Symbiodiniaceae communities between free-living Symbiodiniaceae pools, thus decreasing beta diversity, but that it would not significantly alter coral-associated Symbiodiniaceae communities. Quantifying the composition and variability of Symbiodiniaceae communities in response to local stress under baseline thermal conditions can provide insights into how coral symbioses interact with their dynamic environment, which can inform our understanding of how these critical symbioses may be altered during, or reestablished following, future bleaching events.

## Results

### Overall diversity patterns

**Figure 2:**
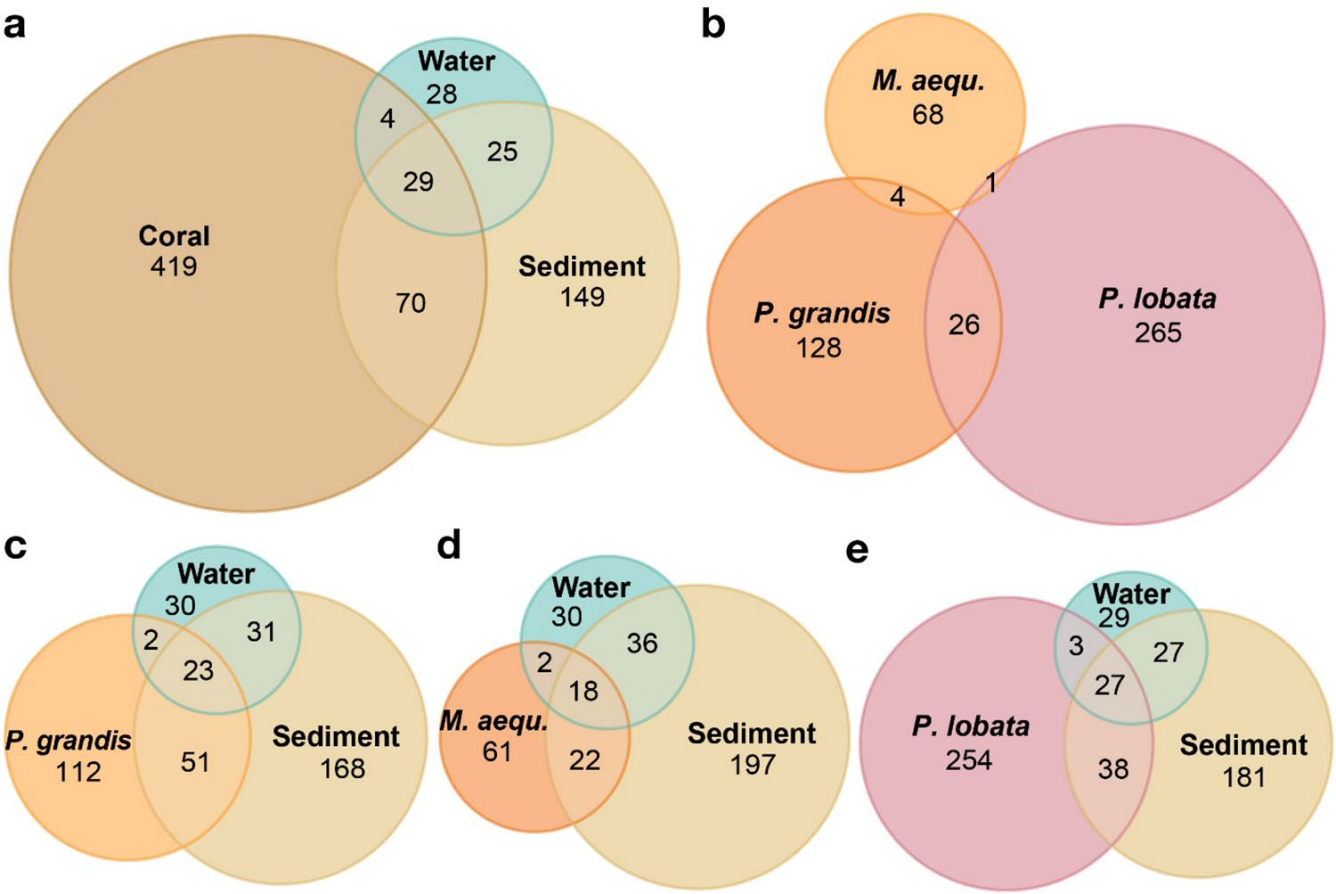
Symbiodiniaceae amplicon sequence variants (ASVs) at all sites and time points combined (**a**) in each type of reef habitat compartment (i.e., coral, sediment, water), (**b**) in each coral species (i.e., *P. grandis, M. aequituberculata* [*M. aequ*.]*, P. lobata*), and in each type of reef habitat compartment, but considering the different coral species individually: (**c**) *P. grandis*, (**d**) *M. aequituberculata* [*M. aequ*.], (**e**) *P. lobata*. Venn diagram shows the number of Symbiodiniaceae ASVs present in each sample type, as well as the amount of overlap between and among sample types.

Symbiodiniaceae sequence identities varied substantially across the three distinct reef habitat compartments sampled (coral, water, sediment; Fig. 2a,c–e), as well as amongst the three coral species (Fig. 2b). In total, we detected 724 amplicon sequence variants (ASVs; coral, 522; sediment, 273; water, 86) from six of the seven named Symbiodiniaceae genera and one unnamed ‘clade’ (*Symbiodinium sensu stricto*, *Breviolum, Cladocopium*, *Durusdinium*, *Fugacium*, *Gerakladium*, and ‘clade I’; Fig. S3). In each reef habitat compartment, there were far fewer dominant ASVs and clades (defined as the most common within a sample; comprising at minimum 25% of the proportional abundance) compared to the total number of each respectively (Table 1).

**Table 1:** Number of dominant and total observed genera and ASVs in each reef habitat compartment.

Reef Habitat Compartment	Number of Dominant Genera	Total Number of Genera	Number of Dominant ASVs	Total Number of ASVs
Sediment	3	6	10	273
Water	3	5	10	86
*M. aequituberculata*	1	6	1	103
*P. grandis*	2	3	1	188
*P. lobata*	1	5	7	322

### Symbiodiniaceae across reef habitat compartments

Despite occurring in close proximity on the reef, only 4% (29) of the Symbiodiniaceae ASVs were found in all three reef habitat compartments, and an additional 14% were found in two of the three reef habitat compartments (Fig. 2a). More than half of all Symbiodiniaceae ASVs (419) were found uniquely in the coral compartments; 21% percent (149) were found uniquely in sediment, and 4% (28) occurred only in water (Fig. 2a). Compared to the two free-living compartments, the coral *P. lobata* had more unique ASVs than either sediment or water (Fig. 2e). *M. aequituberculata* and *P. grandis* had fewer unique ASVs than sediment but more than water (Fig. 2c,d).

No Symbiodiniaceae ASVs were shared amongst all three coral species (Fig. 2b): *P. lobata* had the most unique ASVs (265; 51% of all coral-associated ASVs), followed by *P. grandis* (n = 128; 25% of all coral-associated ASVs) and *M. aequituberculata* (68; 13% of all coral-associated ASVs) (Fig. 2b). *P. grandis* was dominated by one ASV (which blasted to ITS2 C42a), *M. aequituberculata* by a single ASV (which blasted to ITS2 C31), and *P. lobata* by 7 ASVs (which all blasted to C15). Overlapping ASVs amongst the three coral species were either background symbionts in both coral species, or a dominant ASV in one coral species that was a background ASV in the other coral species.

### Chronic local disturbance

**Figure 3:**
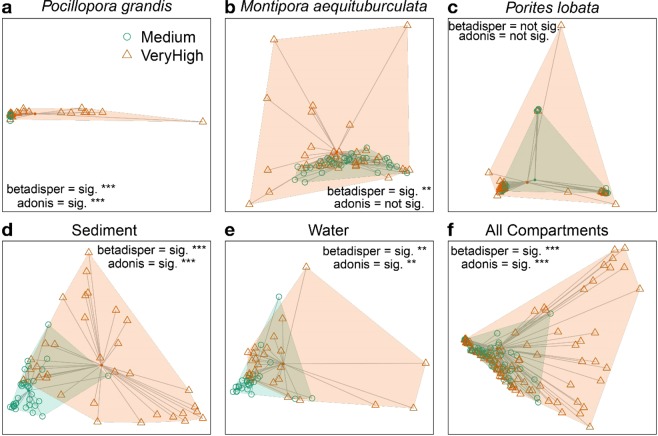
Multivariate ordination (PCoA) of Symbiodiniaceae communities associated with coral (**a**–**c**), free-living (**d**,**e**) samples, and all samples combined. (**f**) Points indicate individual samples (connected to the centroid point in the center), color indicates human disturbance level, and shaded areas indicate boundaries of observed community structure. p-values represent statistics from the beta dispersion analysis. Significance level of each test is denoted by ***<0.001, **<0.01, *<0.05.

There were significant differences in beta diversity between symbiont communities exposed to medium and high chronic disturbance levels for all reef habitat compartments, except for the coral *P. lobata* (Fig. 3). Multivariate location was significantly different between disturbance levels for *P. grandis* (PERMANOVA, F = 12.4, R^2^ = 0.15, p = 0.001; Fig. 3a), sediment (PERMANOVA, F = 23.1, R^2^ = 0.26, p = 0.001; Fig. 3d), and water (PERMANOVA, F = 5.6, R^2^ = 0.10, p = 0.002; Fig. 3e), but not for *M. aequituberculata* (PERMANOVA, p > 0.05; Fig. 3b) or *P. lobata* (PERMANOVA, p > 0.05; Fig. 3c). Multivariate dispersion (i.e., variation) was also greater in the high disturbance sites than in the medium disturbance sites for *P. grandis* (PERMDISP2, F = 46.1, p < 0.001; Fig. 3a), *M. aequituberculata* (PERMDISP2, F = 7.3, p < 0.001; Fig. 3b), sediment (PERMDISP2, F = 100.0, p < 0.001; Fig. 3d), and water (PERMDISP2, F = 9.7, p = 0.003; Fig. 3e) but was not significantly different for *P. lobata* (PERMDISP2, p > 0.05; Fig. 3c). *P. grandis* also showed the most dramatic change in genus-level Symbiodiniaceae community structure with chronic disturbance: at medium disturbance sites *P. grandis* colonies were primarily dominated by *Cladocopium*, with zero or very low abundances of *Durusdinium*, while at the high disturbance level, *Durusdinium* was present in several samples at sequence abundances>20% (Supplementary Fig. S3).

### Pulse disturbance overlaid on two levels of chronic disturbance

**Figure 4:**
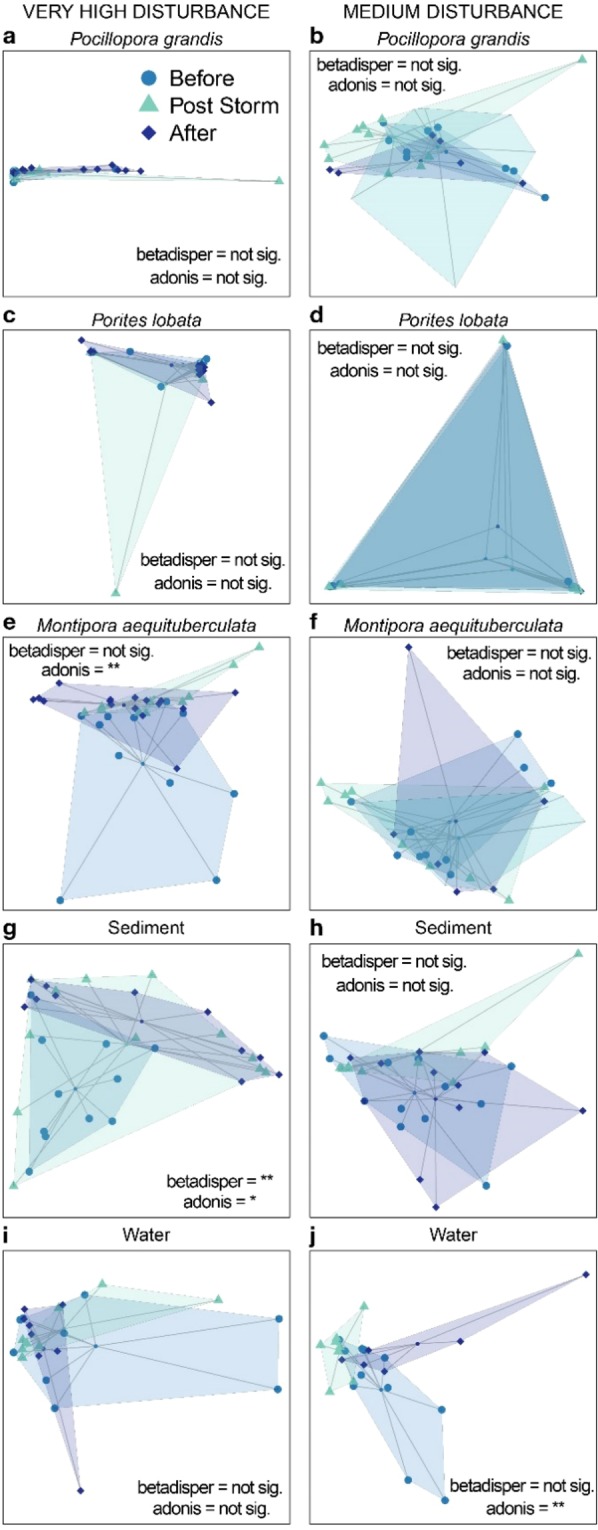
Multivariate ordination (PCoA) of Symbiodiniaceae communities associated with coral (**a**–**f**), sediment (**g**,**h**), and water (**i**,**j**) samples. Points indicate individual samples (connected to the centroid point in the center), color indicates sampling time point, and shaded areas indicate boundaries of observed community structure. Significance level of each test is denoted by **<0.01, *<0.05.

The pulse disturbance had a variable influence on Symbiodiniaceae beta diversity, depending on the reef habitat compartment and the underlying level of chronic disturbance. In the water column, multivariate location differed significantly over time at the medium, but not at the high disturbance sites (Table 2; Fig. 4i,j). In *M. aequituberculata*, the opposite occurred, with evidence of changes in multivariate location over time at the high, but not at the medium disturbance sites (Table 2; Fig. 4e,f). Similarly, in the sediment, there was evidence of changes in both multivariate location and variation over time at the high, but not at the medium disturbance sites (Table 2; Fig. 4g,h). Beta diversity metrics for *P. grandis* and *P. lobata* colonies did not change over time in either disturbance treatment (Table 2, Fig. 4a–d).

**Table 2:** Changes in symbiont beta diversity over time, with reef habitat compartments subjected to a pulse disturbance.

	High Disturbance		Medium Disturbance	
	Multivariate location	Beta dispersion	Multivariate location	Beta dispersion
*P. grandis*	**p > 0.05**	**p > 0.05**	**p > 0.05**	**p > 0.05**
*M. aequituberculata*	F = 3.2 R^2^ = 0.15 p = 0.003	**p > 0.05**	**p > 0.05**	**p > 0.05**
*P. lobata*	**p > 0.05**	**p > 0.05**	**p > 0.05**	**p > 0.05**
Sediment	F = 2.6 R^2^ = 0.14 p = 0.017	F = 7.1 p = 0.003	**p > 0.05**	**p > 0.05**
Water	**p > 0.05**	**p > 0.05**	F = 2.7 R^2^ = 0.20 p = 0.004	**p > 0.05**

Beta dispersion (PERMDISP) and multivariate location (PERMANOVA) over time for each reef habitat compartment. Bold indicates non-significant model results.

**Figure 5:**
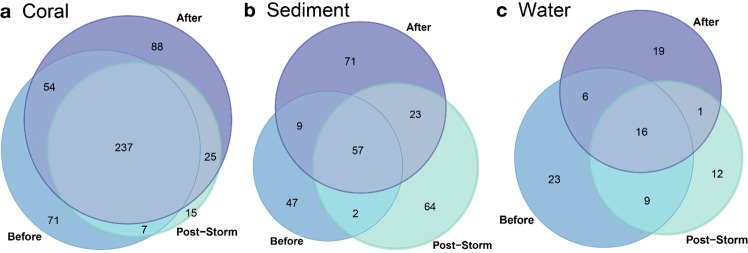
Consistency of Symbiodiniaceae ASVs in each reef habitat compartment before (August 2014), immediately post-storm (January 2015), and after (May 2015) the pulse disturbance caused by the winter storm wave event. Venn diagrams show the number of Symbiodiniaceae ASVs present during each time point, as well as the amount of overlap between and amongst the time points.

Despite some observed differences in symbiont beta diversity across the three sampling time points (Table 2; Fig. 4), much of the symbiont community remained consistent within each reef habitat compartment over the course of this pulse disturbance (Fig. 5). For example, nearly half (48%) of all ASVs present in corals were observed during all three time points (Fig. 5). Sediment and water exhibited less consistency over time, with less than a quarter of ASVs present in all three time points (sediment = 21%, water = 19%; Fig. 5). Only a few ASVs were found in only two time points (up to 11% in any one reef habitat compartment), and a substantial number of ASVs were observed during only one time point (coral = 35%, sediment = 67%, water = 63%).

## Discussion

A fundamental ecological question is whether disturbance increases beta diversity by allowing for habitat expansion by a suite of background or potentially opportunistic symbionts (in accordance with the Anna Karenina principle), or whether it decreases beta diversity due to environmental filtering or biotic homogenization. Thus far, the former mechanism appears to be more common in microbial systems, with elevated beta diversity with disturbance documented in multiple ecosystems, including soil bacteria in the tropics^54,55^, and animal microbiomes^17^, including those of corals^14,16^. Our study provides further evidence of the Anna Karenina principle, and supports our hypothesis that local human disturbance is associated with consistent increases in beta diversity (i.e., variation, and to a lesser extent turnover) across Symbiodiniaceae communities in most reef habitat compartments. This means that there was an increase in the number of potential Symbiodiniaceae community compositions in both endosymbiotic and free-living communities, and that individual samples were more different from one another at highly disturbed sites compared to moderately disturbed ones. These findings are in accordance with a previous study on the Great Barrier Reef which found that background levels of Symbiodiniaceae were greatest in locations with poor water quality^56^. Increased symbiont community variation has been associated with high mortality in the larval and juvenile stages of one coral species (*Acropora tenuis*)^19^, although less is known about adult coral growth and survival.

Contrary to our second hypothesis, despite some variability among sampling time points, we detected very few changes in the Symbiodiniaceae sequence communities within or among chronic disturbance treatments in response to a pulse disturbance event (Fig. 4, Table 2). However, there was some variability over time, raising questions about whether these changes are due to stochasticity in Symbiodiniaceae populations, or whether site-specific environmental drivers might be at play. Consistent with previous studies, we found that *Porites lobata* symbioses are highly specific (Table 2, Fig. 4), dominated by *Cladocopium* C15-like ASVs, and stable across both time and space^57,58^.

Although there has been a recent increase in the use of next-generation sequencing techniques for quantifying *in hospite* Symbiodiniaceae communities^59–61^, the use of these methods for Symbiodiniaceae identification in other reef habitat compartments is still rare (but see^28,58,62^). We add to this nascent literature here, expanding our understanding of spatial and temporal variability in Symbiodiniaceae communities, and how they vary under the influence of multiple stressors. In contrast with two recent studies on the Great Barrier Reef (GBR) that found four times more unique operational taxonomic units (OTUs) in sediments than in juvenile corals^28^ and two times more unique OTUs in water and sediment than in adult corals^62^, we found 2.8-fold more unique ASVs in adult corals than in sediment, and 2.4-fold more unique ASVs in adult corals than in water and sediment combined. From the host perspective, it is possible that the difference in the number of ASVs in coral and sediments between these studies was due to the focal coral species and life stages sampled in each study. However, we view this as unlikely because although Quigley *et al*. (2017) sampled juveniles of two closely related Acroporids (*A. tenuis* and *A. millepora*), Ziegler *et al*. (2018) sampled a range of adults from endosymbiotic hosts that encompassed more diversity than our study. Conversely, from the symbiont perspective, it is possible that there are fewer free-living Symbiodiniaceae on Kiritimati than on the Great Barrier Reef, due to the isolation of this remote atoll. While symbiotic Symbiodiniaceae could potentially arrive on Kiritimati in association with coral larvae with long planktonic larval durations, connectivity of free-living Symbiodiniaceae from reefs several hundred to thousands of kilometers away may be limited^63,64^. This idea is supported by previous research demonstrating that Symbiodiniaceae tend to disperse locally^65,66^ and endosymbiotic Symbiodiniaceae are competent in the water column for only approximately seven days^67^, while coral larval competency can be one month or more^68–70^.

Despite the discrepancy between symbiotic and free-living Symbiodiniaceae diversity observed between these studies^28,62^, the proportion of ASVs shared between coral and sediment in them was similar (9.7% of ASVs excluding those found only in water in our study, compared to 10.6% of OTUs shared between sediment and juvenile corals in^28^). However, both our study and Quigley *et al*.^28^ found different proportions of shared ASVs and OTUs than Ziegler *et al*.^62^, which found that half of all environmental Symbiodiniaceae OTUs were also found in symbiosis, although this may in part be due the fact that they surveyed symbioses of not only scleractinian corals but also soft corals and Foraminifera. Future research on Symbiodiniaceae connectivity and diversity on a variety of reefs across a broad geographic range may help to reveal how environmental and biological drivers shape patterns of Symbiodiniaceae survival and persistence in both symbiotic and free-living environments on a reef.

Although we consider all Symbiodiniaceae found in the sediment to be free-living (either transiently or exclusively), some Symbiodiniaceae also occur in symbiosis with benthic Foraminifera (e.g., *Cladocopium* C91) that may be captured during sediment collection. It is possible that even after pre-filtering at 20 µm (which should remove all living symbiotic benthic forams), mechanical damage could have broken open forams and released endosymbiotic algae that were then detected as part of the sediment-associated free-living community. We expect that this represents a small amount of the sequences extracted from the sediment, but future work could focus on comparisons between endosymbiotic Symbiodiniaceae in forams and free-living Symbiodiniaceae from the same sediment samples. Relatedly, a previous study showed that foram assemblages on Kiritimati have changed in response to chronic human disturbance over the past 40 years, towards heterotrophic rather than symbiotic assemblages^71^, which may influence the abundance and community structure of foram-associated Symbiodiniaceae by decreasing populations of their foram hosts. Additionally, foram-associated Symbiodiniaceae may participate in driving the evolution of coral-associated Symbiodiniaceae^72^, so further research on the exchange of symbionts among foraminiferans, sediment, and corals may reveal ecological and evolutionary connectedness among these major reef habitat compartments.

Although overall Symbiodiniaceae community structure varied amongst the three coral species, and no ASVs were shared among all three coral species, there was overlap in the presence of 31 Symbiodiniaceae ASVs between two of the three coral species, which may provide reservoirs of symbiotically competent Symbiodiniaceae cells. All of the ASVs that were shared between coral species were background symbionts in at least one of the two species, since each species was dominated by only a small number (1–7) of ASVs in each coral species. Since each ASV is a unique sequence, we expect that some of these ASVs may be intragenomic variants of Symbiodiniaceae species. For example, we consider it likely that the 7 ASVs that blasted to *Cladocopium* C15 are likely IGVs of one (or maybe a couple) of C15 (or C15-like) species, as opposed to 7 different *Cladocopium* C15-like species. However, our data and analyses cannot address this question directly. Dominance by a small number of ASVs among colonies of each coral species corresponds with previous research showing that nearly all corals host background symbionts^73^ and that background communities can change significantly even when the dominant symbionts remain stable^74^. Debate is still ongoing regarding how much impact these types of background symbionts have on holobiont ecology^45,75^.

In this study, we focused on three coral species common on Kiritimati reefs that are vertical transmitters of Symbiodiniaceae (Symbiodiniaceae propagules are passed from parent colonies to offspring, limiting the necessity for these species to uptake Symbiodiniaceae from the environment)^35,76,77^. However, recent research has shown that Symbiodiniaceae uptake by larvae may still be important, even for vertical transmitters^78,79^. Additionally, since there is substantial overlap in symbiont communities among these three phylogenetically disparate, vertically transmitting coral species, it is likely that there is also overlap with other species that rely primarily on horizontal uptake of symbionts each generation. This may be due to regional adaptation on Kiritimati (e.g., dispersal limitation or environmental filtering), but additional research is necessary to evaluate this hypothesis.

Measuring changes in Symbiodiniaceae communities at varying levels of local human disturbance is an important aspect of understanding coral reef resilience, since coral reproduction and recruitment are vital parts of coral recovery after severe bleaching events, and Symbiodiniaceae uptake by juvenile corals is selective within the available pool of symbionts (i.e., bipartite^28^). Although elevated beta diversity may be harmful to coral juvenile survival^19^, there is currently no empirical evidence demonstrating whether elevated beta diversity in Symbiodiniaceae meta-communities is beneficial or harmful to adult coral health and survival. We provide evidence that Symbiodiniaceae beta diversity is greater at sites exposed to higher levels of disturbance, and posit three hypotheses regarding the implications of this increased community variability: elevated beta diversity could be beneficial, because it may offer more combinations of symbionts to the coral host and thus may increase the potential for acclimatization or adaptation of the holobiont^80–82^. This hypothesis should hold true when an increase in symbiotic “options” increases the probability that a coral can take up symbionts which are both good partners and well adapted to the changing ambient environment. Conversely, elevated beta diversity could be harmful, because it may allow for the proliferation of more opportunistic Symbiodiniaceae assemblages that stray towards parasitism by inadequately provisioning their coral hosts^83^. This hypothesis has been widely supported in the general symbiosis research, which suggests that mixing symbionts within a host is typically detrimental to host fitness due to competition among symbionts^84^. However, it is also possible that implications of elevated Symbiodiniaceae beta diversity are context dependent. For example, if elevated Symbiodiniaceae beta diversity introduces opportunistic, heat-tolerant symbionts (e.g., *Durusdinium*), it could benefit the coral host under moderate heat stress (as these symbionts have a greater capacity to resist bleaching) but negatively affect it under baseline conditions (as these symbionts provide less nutrition to their coral hosts). These three hypotheses (the effect of elevated beta diversity is 1) beneficial, 2) harmful, or 3) context dependent) should be further investigated with assays of coral performance in the field and in the lab.

Additionally, our approach warrants further research to verify whether the patterns presented here are consistent across other reef environments in different geographic regions. Future research would need to quantify differences in Symbiodiniaceae community structure across the spectrum of local human disturbance at many different reef locations and for more coral taxa. Our research focused on a small number of sites, clustered by disturbance level, and additional research is needed to expand the geographic range of sites, as well as to test these hypotheses in other locations. While our study found no consistent change amongst reef habitat compartments across three sampling dates, assessment of seasonal changes in Symbiodiniaceae community structure would necessitate additional studies including increased sampling (i.e., multiple years during the same month) to determine whether observed community changes are stochastic, seasonal, or driven by other cyclic or irregular environmental or biological drivers.

In conclusion, we provide empirical evidence that chronic disturbance can increase Symbiodiniaceae beta diversity across reef habitat compartments, while the effect of pulse physical disturbance varies by reef habitat compartment and coral species. If elevated beta diversity is consistent in locations with high levels of local human impact, future studies should investigate whether increased Symbiodiniaceae community variability bolsters or erodes a reef’s capacity for resilience to future stressors.

## Methods

### Study design

We collected coral, water, and sediment samples from four sites on the fore reef (all at 10–12 m depth; Fig. 1, Supplementary Table S1) of Kiritimati (Christmas Island, Republic of Kiribati), a large atoll in the central equatorial Pacific (01 °52′N, 157 °24′W) in August 2014, January 2015, and May 2015. Two of the sites (VH1 and VH3; named as part of a broader long-term monitoring program) experience high human disturbance in the form of localized fishing pressure, sedimentation and pollution due to pier infrastructure near VH1, and localized sewage runoff from adjacent villages, in comparison to the other two sites (M1 and M2), which experience only a moderate level of disturbance, primarily from fishing, and have no infrastructure (Fig. 1; Supplementary Fig. S1 ^85,86^). We quantified the intensity of chronic local human disturbance at each site, using spatial data for two separate metrics, human population densities and fishing pressure (Supplementary Table S2). Specifically, we first generated a geographic buffer (in ArcGIS) to determine human population size within 2 km of each site. Nearly all individuals live in villages, and village location was mapped based on published field surveys^86^. Population size for each village was extracted from the 2015 Population and Housing Census from the Kiribati National Statistics Office^87^. This population metric serves as a proxy for immediate point-source inputs from villages into the marine environment, including pollution and sewage runoff, since Kiritimati does not have any form of wastewater treatment. Secondly, we generated a kernel density function with ten steps based on mapped fishing intensity from^86^. This metric accounts for the more diffuse, but still important, effects of subsistence fishing on the reef ecosystem. We weighted each metric equally, and from this combined metric we grouped the forty sites from our broader ecological monitoring program into five disturbance categories of relative local disturbance (very low, low, medium, high, and very high) (Supplementary Fig. S1 ^85,86^). We selected the four sites for this study because they encompass two distinct levels of local disturbance but have similar physical and oceanographic conditions (Supplementary Table S3), with all four located along the western side of the atoll, along the lagoon face (Fig. 1; Supplementary Fig. S1). We acknowledge that our two medium disturbance sites and our two very high disturbance sites are closer to one another than they are to sites from the other disturbance level. We have assessed local conditions for the sites in each disturbance level (Supplementary Table S3) and conclude that the major factor differing between these two disturbance levels is local human disturbance, rather than prevailing oceanographic conditions (Supplementary Fig. S1). However, we recommend caution regarding broad generalization of these results, and suggest that additional research should address whether these patterns also exist in other locations.

### Wave height

Large swell from a major storm that hit the leeward (western) side of the atoll in January 2015 greatly impacted the reefs at all four sites. We collected a small subset of coral samples two days before the storm disturbance but were unable to collect water or sediment samples immediately before the arrival of the storm waves. Water, sediment, and coral samples were collected from all four sites within two days of the storm, after the waves had subsided enough to allow diving, but while the water was still well-mixed and turbid from the storm (Supplementary Table S4, Table S5).

We quantified the intensity of storm waves during this time using NOAA’s Multi-grid Wavewatch III Hindcast (Supplementary Fig. S2 ^88,89^). Since seasonality is minimal at our equatorial field site, such storm waves generated in the western Pacific may be one of the main drivers of intra-annual temporal variability in Symbiodiniaceae exchange among reef habitat compartments in this region. First, significant wave height and wave direction were extracted for Kiritimati from February 2005 to December 2017. To visualize the wave climate on Kiritimati, a histogram of significant wave heights for the available time period of wave data (~12 years; Supplementary Fig. S2a) and a wave rose (Supplementary Fig. S2b) were plotted. This wave rose shows the primary wave direction (polar coordinates), as well as the distribution of significant wave heights coming from each direction. Waves on Kiritimati primarily come from the E-NE, consistent with the forcing of the trade winds. Waves also occasionally come from the N-NW or the S-SW; these generally represent storm waves and swell coming from the western Pacific.

To determine the severity of the January 2015 storm waves in the context of the broader wave climate on Kiritimati, we extracted the maximum wave height during this event (significant wave height = 2.94 m). Next, a three-parameter generalized extreme value (GEV) distribution was fit to the significant wave height data using the R package extRemes^90^. GEV parameters were fit as follows: location = 1.784 ± 0.002 s.e.; scale = 0.345 ± 0.001 s.e.; and shape = −0.151 ± 0.003 s.e. Next, the R package EnvStats^91^ was used to model the fitted GEV distribution and calculate the percentile of the GEV at the maximum wave height during this event (wave height = 2.94 m, 94th percentile of significant wave height at this location). This allows us to define this storm wave event as a sizeable, but not unprecedented, storm wave event on Kiritimati.

### Sample collection and processing

To characterize Symbiodiniaceae community composition, we collected coral tissue samples from *Pocillopora grandis* (n = 70; previously *Pocillopora eydouxi*)*, Montipora aequituberculata* (n = 78), and *Porites lobata* (n = 73), along with water (n = 50) and sediment (n = 67) samples (Supplementary Table S1). We sampled coral colonies along a 60 m transect at each site, using a small metal chisel to biopsy (<1 cm^3^) small tissue samples of *P. lobata* and *M. aequituberculata* and by breaking off a small branch of *P. grandis*. Each sample was immediately transferred to an individual sterile sample bag (WhirlPak® Sterile Sampling Bags), and upon surfacing from the dive stored in a cooler on ice until laboratory processing began in the evening. We collected water samples (2.98–10.95 L seawater per sample, mean = 7.0 L, s.d. = 1.6 L; Supplementary Table S4) within 1 m of the reef surface. The water sample volume varied among samples due to field conditions as well as filter capacity. We collected sediment samples (2–7 mL per sample; Supplementary Table S5) along the same transect as the coral colonies (i.e., <1 m distance from the transect tape), sampling the top layer of sediment within 3 cm of the water-sediment interface, which was generally loose and well-mixed.

During processing, we cleaned coral biopsies using filtered freshwater to remove as much skeleton as possible, and approximately 50 µL of coral tissue was preserved in 400 µL of guanidinium buffer (50% w/v guanidinium isothiocyanate; 50 mM Tris pH 7.6; 10 µM EDTA; 4.2% w/v sarkosyl; 2.1% v/v-mercaptoethanol). *P. grandis was* scraped with a pick and razor to dislodge the coral tissue before being placed in the guanidinium buffer. We cleaned all tools with thorough freshwater rinse, followed by a KimWipe with ethanol, and a dry KimWipe in between each sample. All samples were stored at 4 °C until DNA extraction. To process the sediment samples, we resuspended each sample in 350–400 mL filtered seawater (0.22 µm) and filtered them using a two-step process. First, the resuspended sediments were gravity filtered through a 20 µm pore size nylon mesh filter, and the resulting solids were discarded. Secondly, the filtrate was passed through a Whatman Nucleopore track-etched 5 µm pore size filter. Each 5 µm filter and its contents was preserved in a 1.5 µL centrifuge tube with 400 µL of guanidinium buffer, and the filtrate was discarded. We filtered the water samples using the same two-step filtering process and preserved them the same way. Quantities of water and sediment filtered for each sample are listed in Tables S4 and S5. DNA was extracted from all samples using a guanidinium-based extraction protocol^92,93^, modified to include three 70% ethanol washes (rather than one). After extraction, DNA was cleaned using Zymo Genomic DNA Clean and Concentrator^TM^ -25 (Catalog Nos. D4064 & D4065) to improve downstream processing (http://www.zymoresearch.com/downloads/dl/file/id/638/d4064i.pdf).

Samples were prepared for ITS2 amplicon sequencing on the Illumina MiSeq platform. Library preparation and Illumina MiSeq ITS2 amplicon sequencing was performed by the Hawaii Institute of Marine Biology (HIMB) Genetics Core Lab following the Illumina 16 S Metagenomic Sequencing Library Preparation (Illumina protocol, Part # 15044223 Rev. B) with modifications to generalize this protocol for ITS sequences. ITS primers (ITS-forward and ITS-reverse^92^) were used instead of the 16 S primers. Additional modifications included: PCR 1 annealing temperature was changed to 52 °C, 60 µl of SPRI beads were used for PCR 1 clean up, and PCR 1 bead clean up elution buffer volume varied depending on the Qubit concentration of initial gDNA (concentrations with ≤1 ng/µl were resuspended in 12.5 µl elution buffer; 2–4 ng/µl were resuspended in 42.5 µl elution buffer; and ≥5 ng/µl were resuspended in 52.5 µl elution buffer). Prepared libraries were sequenced on the Illumina MiSeq platform with 2 × 300 paired end read chemistry.

In Symbiodiniaceae, ITS2 is a multi-copy marker that exhibits intragenomic variation (IGV)^94^. Despite the presence of IGV, this marker is widely used to characterize Symbiodiniaceae sequence assemblages, but approaches must be carefully considered in this light^95^. We avoid a traditional operational taxonomic unit (OTU) approach in our analysis, as this can collapse IGVs into incorrect groupings since IGVs may not correspond to the typical 97% groupings (i.e., sequences that are IGVs may be more than 3% different from one another, while sequences that come from distinct Symbiodiniaceae species may be less than 3% different from one another). Instead, we utilize an ASV (amplicon sequence variant) approach, detailed below. Each ASV represents a unique sequence, and therefore assumes no phylogenetic relatedness based on sequence similarity, limiting potential mischaracterizations of sequence diversity. Consequently, our approach does not directly address species diversity of Symbiodiniaceae, but rather addresses differences in sequence diversity among samples.

### Bioinformatics

All code for this bioinformatics pipeline is available on GitHub (https://github.com/baumlab/Claar_etal_2020_SciRep/). Raw sequences were quality controlled, merged, filtered, and assigned taxonomy using the DADA2 ITS pipeline^96^. This pipeline produces amplicon sequence variants (ASVs), which are quality-controlled exact sequence variants. The DADA2 pipeline removes spurious sequences, including chimeras, and retains real sequence variants from the sampled community^96^. Resulting ASV tables, taxonomic information, and sample metadata were analyzed using the *phyloseq* package^97^ implemented in R^98^. A total of 338 samples were successfully amplified and sequenced (coral n = 221; sediment n = 67; water n = 50; Supplementary Table S1), yielding a final dataset consisting of 4,840,494 sequences. Finally, a phylogenetic tree (similar to^57^) was built by separating Symbiodiniaceae sequences by genus (previously clade-level) identity. Sequences were aligned within each clade separately (AlignSeqs from R package DECIPHER 2.12.0^99^). A distance matrix among Symbiodiniaceae genera was created based on nr28s-rDNA distances^100^, and upgma (R package phangorn v.2.2.0^101^) was used to build a representative phylogenetic tree based on between clade distances (nr28s) and within-clade alignments (ITS2) that was incorporated into the phyloseq object.

### Statistics and visualization

We used multivariate statistics and visualization to examine variability in Symbiodiniaceae beta diversity: (1) between sites with distinct chronic disturbance levels (for each individual reef habitat compartment), and (2) over time (i.e., across a pulse disturbance), for each reef habitat compartment and disturbance level. For each question, we first assessed the community structure of Symbiodiniaceae sequence assemblages, using a permutational multivariate analysis of variance (PERMANOVA, global, n = 999 permutations; *adonis* function in the R package vegan^102^). This analysis assesses community structure as a combination of multivariate location (“turnover”) and variation. Second, we evaluated ‘variation’ by quantifying changes in multivariate dispersion using PERMDISP (*betadisper* function; vegan). To display the variation of Symbiodiniaceae communities at each sampling time point, we conducted PCoA analysis with the *betadisper* function (in vegan), using weighted UniFrac distances. Weighted UniFrac is a phylogenetically informed beta diversity distance metric that considers relative abundances of each ASV and is useful for examining differences in community structure in environmental samples^103^.

Disentangling turnover from multivariate dispersion can be problematic when the PERMANOVA is significant and groups have significantly different multivariate dispersions (i.e., PERMDISP is significant)^104^. However, PERMANOVA is robust to heterogeneous dispersions if the design is balanced (i.e., the same number of samples in each group being tested)^104^. Therefore, we conducted a bootstrapped sensitivity analysis of the PERMANOVA (adonis) results from each reef habitat compartment that showed significant results for both PERMANOVA and PERMDISP. To do this, we randomly subsampled the larger sample number to the smaller sample number (e.g., for *P. eydouxi*, original n_(Medium)_ = 39, n_(VeryHigh)_ = 31, subsampled to n = 31 in each group; Supplementary Table S6). We bootstrapped this sensitivity analysis 100 times, and if results remained significant for all iterations, we concluded that our original results were robust to slight deviations from a balanced sample design and that a significant PERMANOVA result really did indicate turnover.

We used the Eulerr package in R^105^ to create Venn diagrams to visualize shared Symbiodiniaceae ASVs among reef habitat compartments and over space and time. We utilized the iNEXT^106^ package to quantify and visualize ASV accumulation curves in order to visualize the relationship between sampling depth and number of ASVs observed. Three curves were generated for each reef habitat compartment (coral, sediment, water): by disturbance level, by site, and by field season (Supplementary Figs. S4–S6). Curves generally reached an asymptote at the sampling depth (with the exception of water at site VH1), indicating that sequencing depth was sufficient to capture the full Symbiodiniaceae community at these aggregated levels. Finally, ggplot2^107^ was used for visualizing our results. All analyses were conducted in R version 3.6.0, and all code is available on GitHub.

## Supplementary information

Supplementary Materials.

## Data Availability

Data reported in this paper and code to reproduce these analyses are provided in the supplementary materials or at https://github.com/baumlab/Claar_etal_2020_SciRep/ (10.5281/zenodo.3678916), and sequences are available on Zenodo (10.5281/zenodo.3678934).
