# Supplementary material for: Chronic disturbance modulates symbiont (Symbiodiniaceae) beta diversity on a coral reef
Source: Sci Rep. 2020 Mar 11;10:4492. doi: 10.1038/s41598-020-60929-z (PMC7066189; doi:10.1038/s41598-020-60929-z)
Supplement: Supplementary file 1 — Supplementary Materials. [file 41598_2020_60929_MOESM1_ESM.docx]

**Title:** Chronic disturbance modulates symbiont (Symbiodiniaceae) beta diversity on a coral reef

**Authors:** Danielle C. Claar^1*^, Kristina L. Tietjen^1^, Kieran D. Cox^1,2^, Ruth D. Gates^3^, Julia K. Baum^1,3^

1 Department of Biology, University of Victoria, Victoria, British Columbia, V8W 2Y2, Canada.

2 Hakai Institute, Calvert Island, British Columbia, Canada

3 Hawaii Institute of Marine Biology, Kaneohe, HI 96744, USA

**Supplementary Materials**


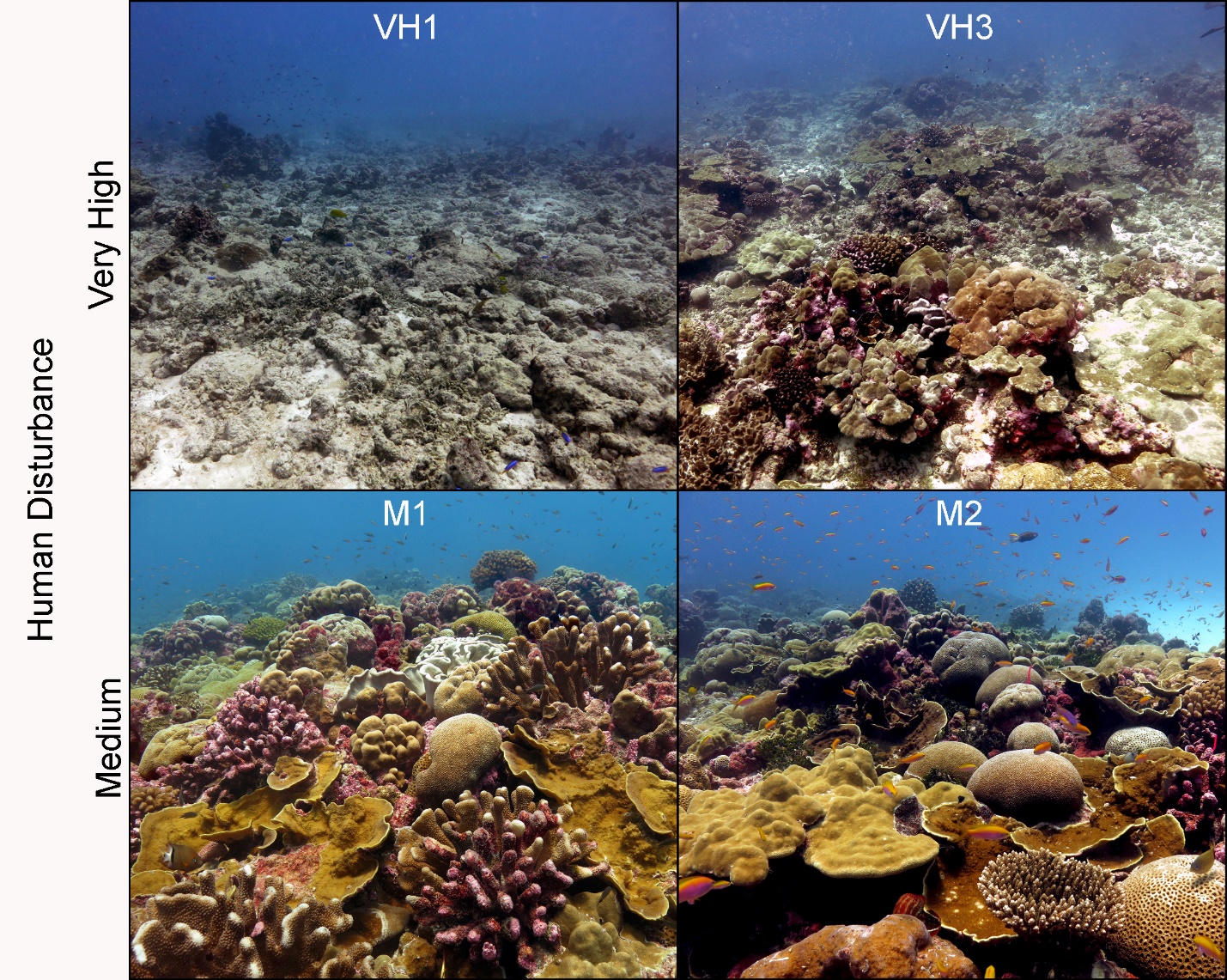


**Supplementary Figure S1. Images of sites exposed to different levels of relative local human disturbance (*very high*, *medium*) on Kiritimati that were included in this study.** Note: Sites are part of a larger ecological monitoring program that includes sites with very high, high, medium, low, and very low levels of disturbance.


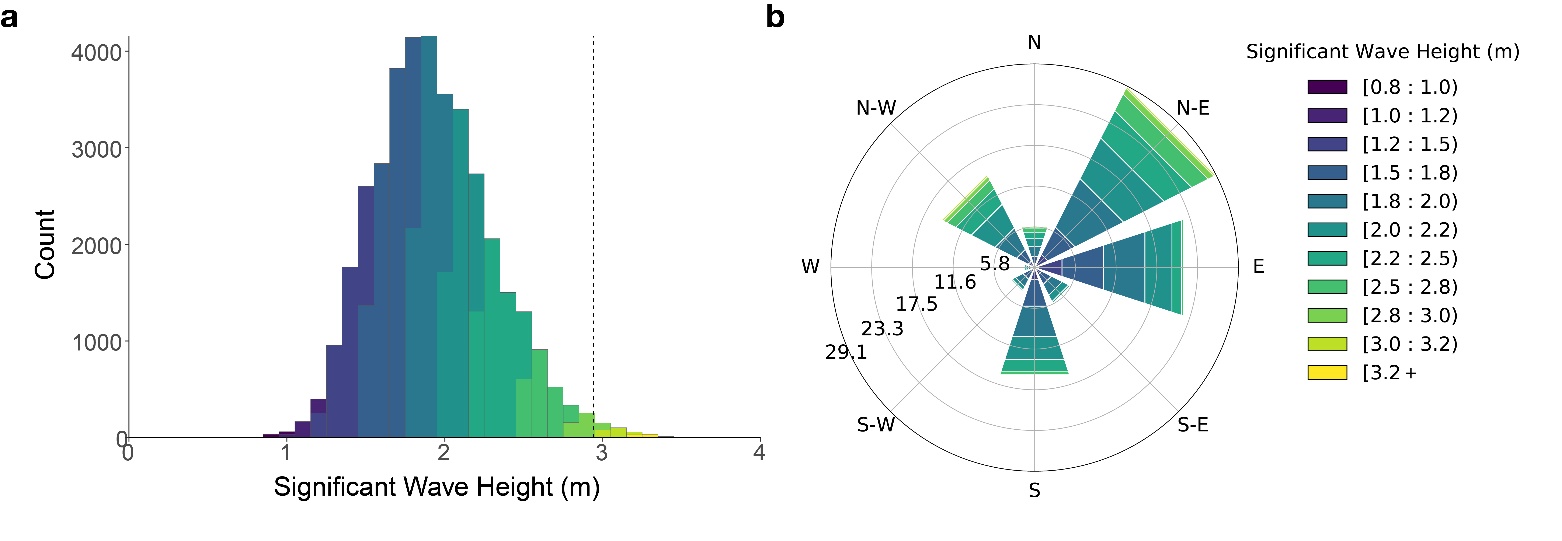


**Supplementary Figure S2. Wave climate on Kiritimati (2005-2017) extracted from NOAA’s Wavewatch III hindcast product.** Plots are based on 3-hourly wave models. **a** Histogram of significant wave height. Colors indicate significant wave height, and dashed line on panel a indicates maximum wave height on Kiritimati in January 2015 (2.94 m). **b** Polar rose showing average wave conditions. Each bin represents 45° increments. Colors indicate significant wave height, angle of each bin indicates direction of incoming waves, and distance from center represents the probability (percentage) of waves coming from each direction.

**Supplementary Figure S3. Bar plots of Symbiodiniaceae taxa, colored by genus in the different reef compartments and coral species**: **a** sediment, **b** water, **c** *Montipora aequituberculata,* **d** *Pocillopora grandis,* **e** *Porites lobata.*


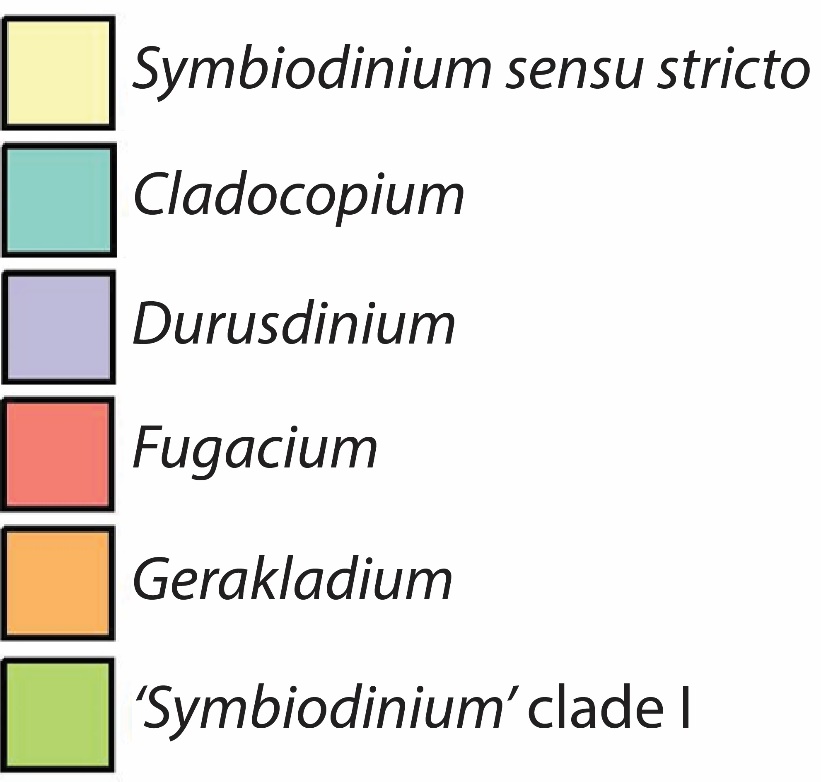


Supplementary Figure S3. **a** Sediment
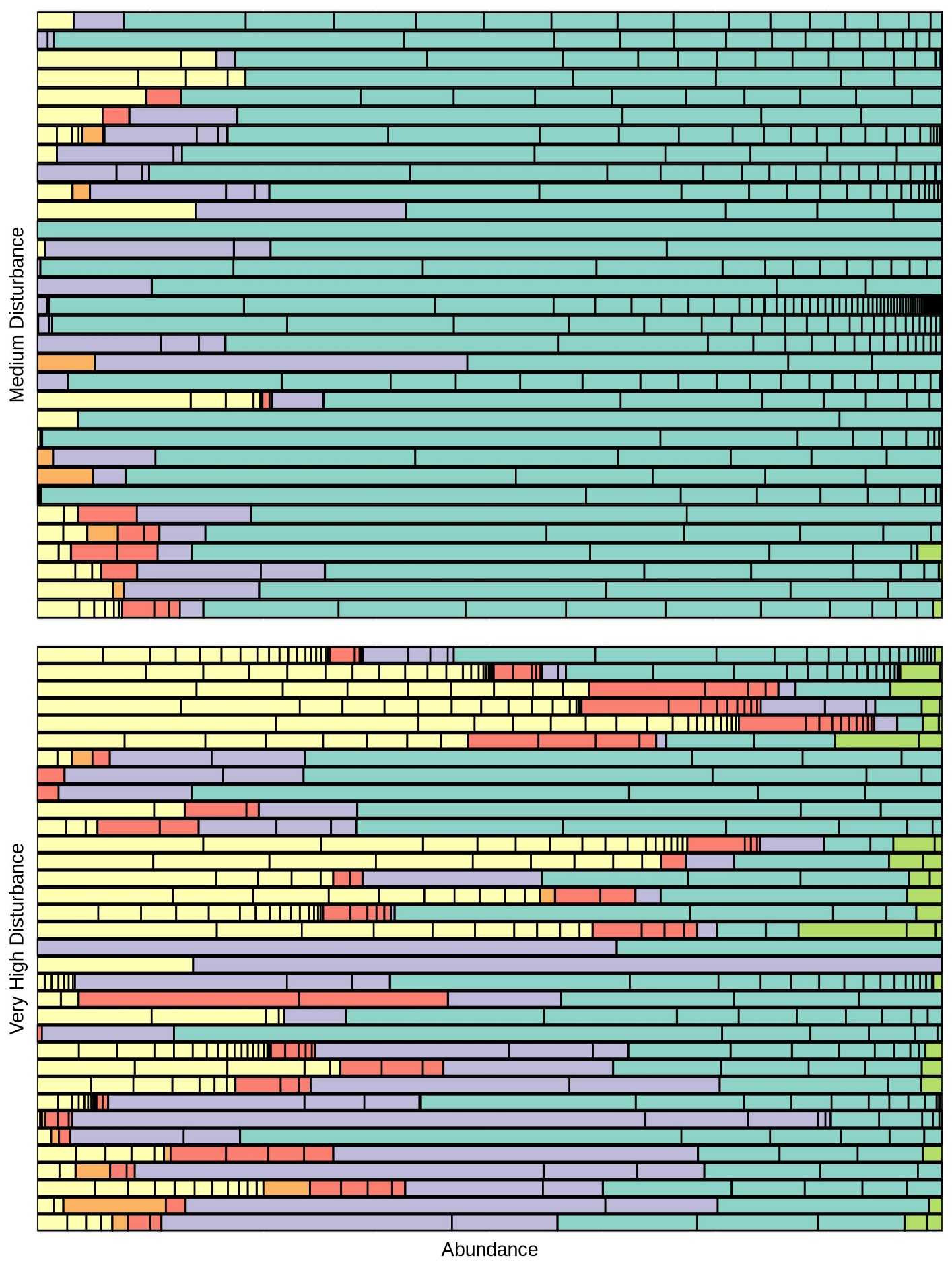


Supplementary Figure S3. **b** Water


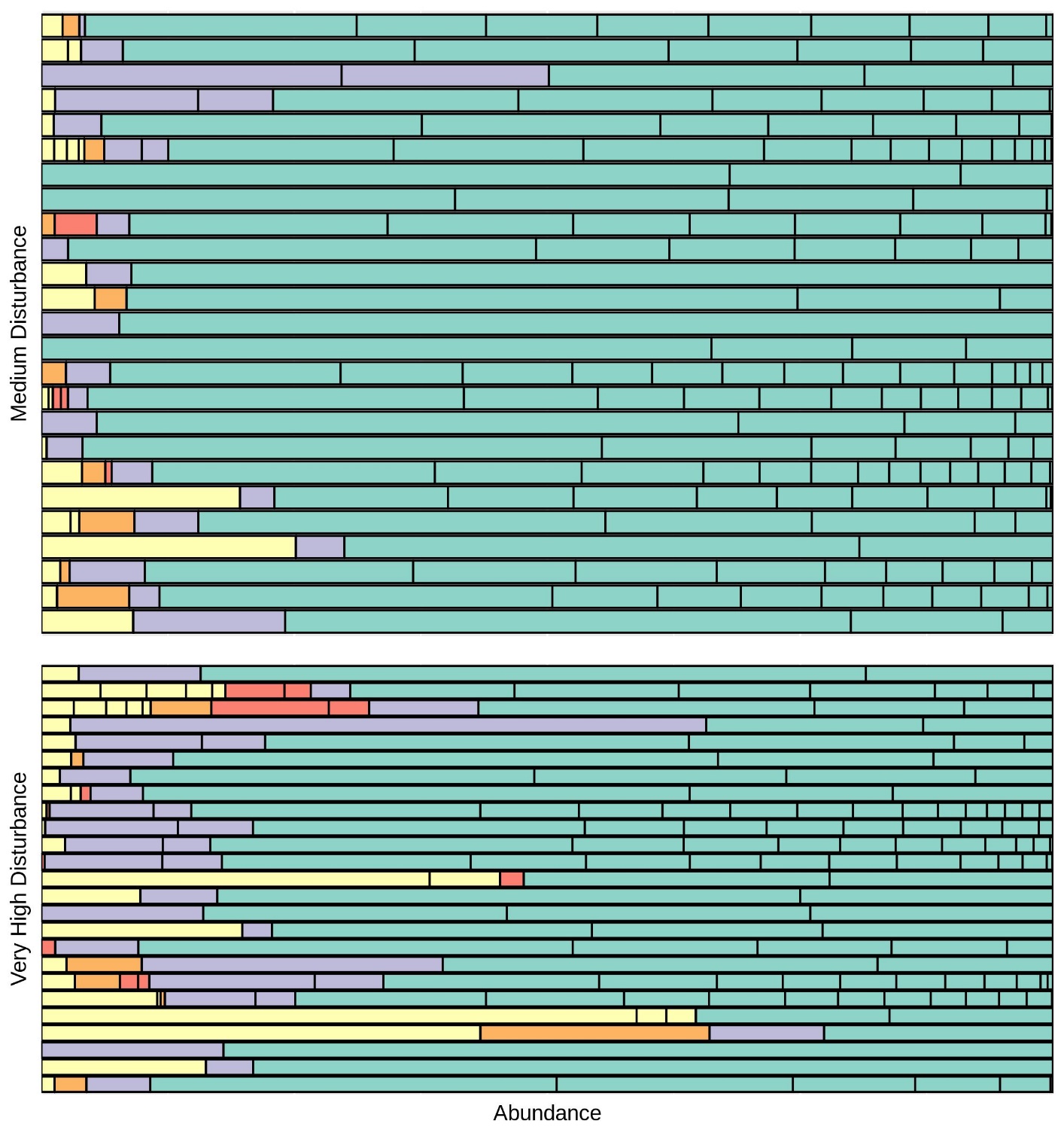


Supplementary Figure S3. **c** *Montipora aequituberculata*


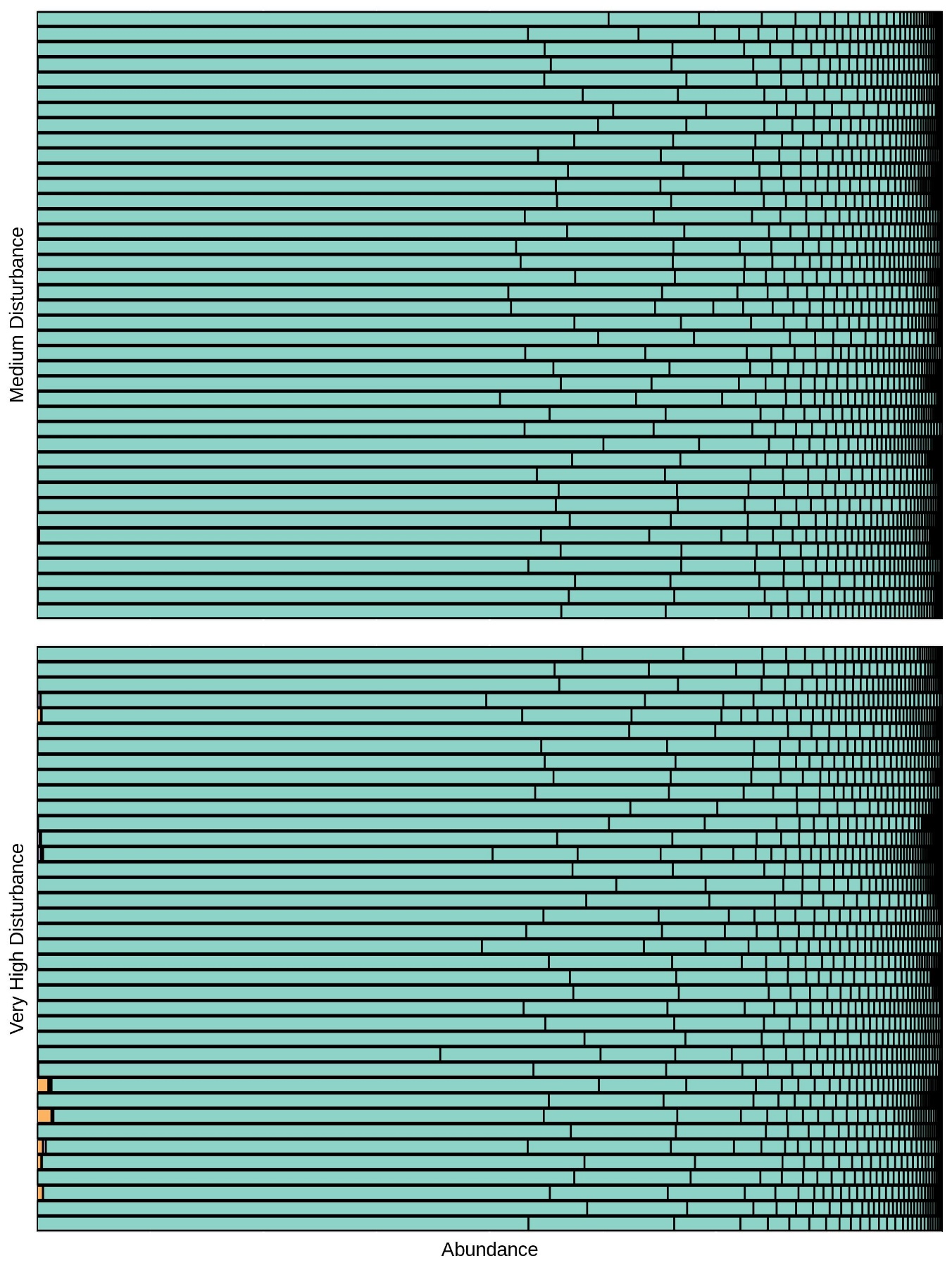


Supplementary Figure S3. **d** *Pocillopora grandis*


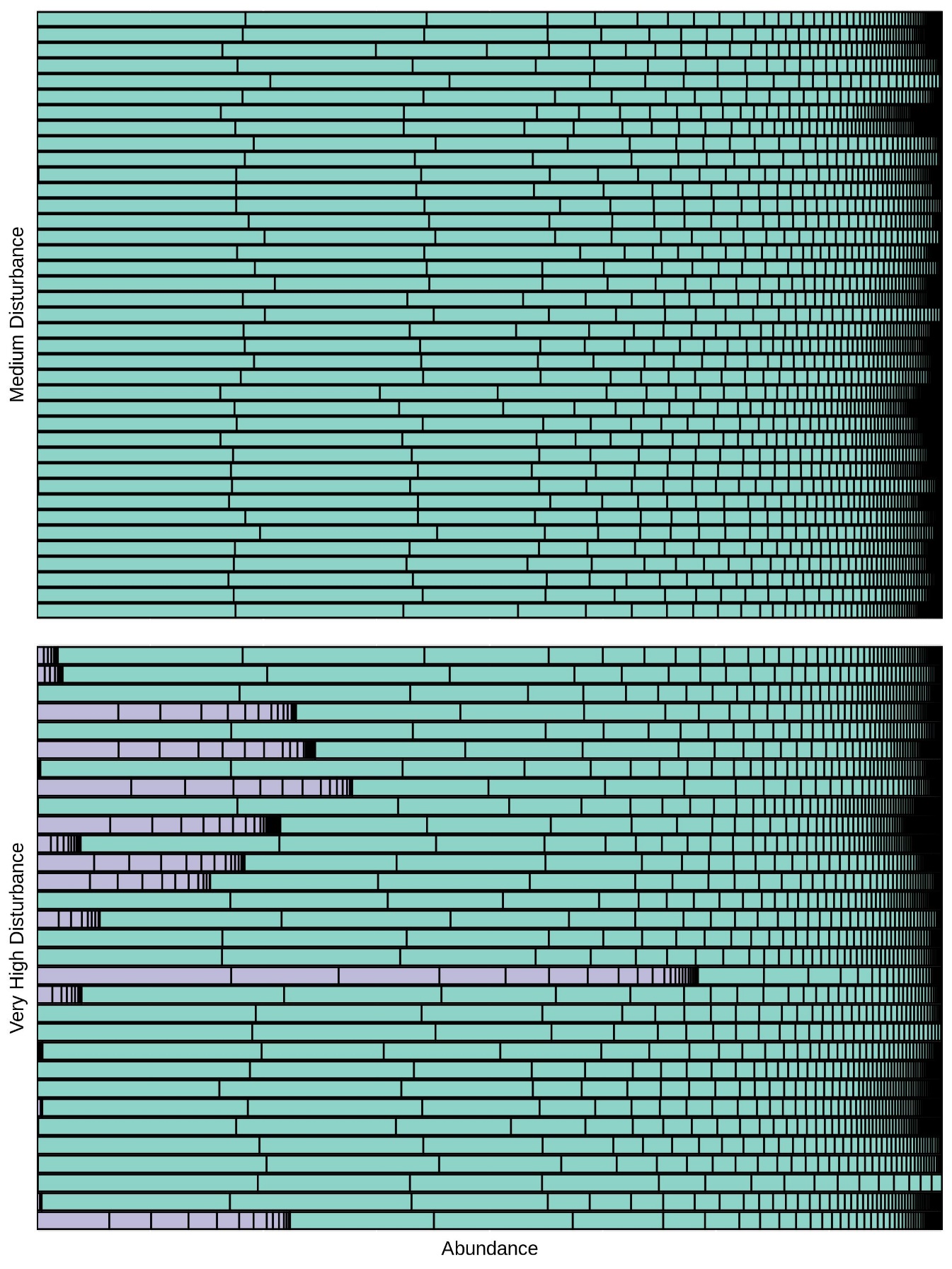


Supplementary Figure S3. **e** *Porites lobata*


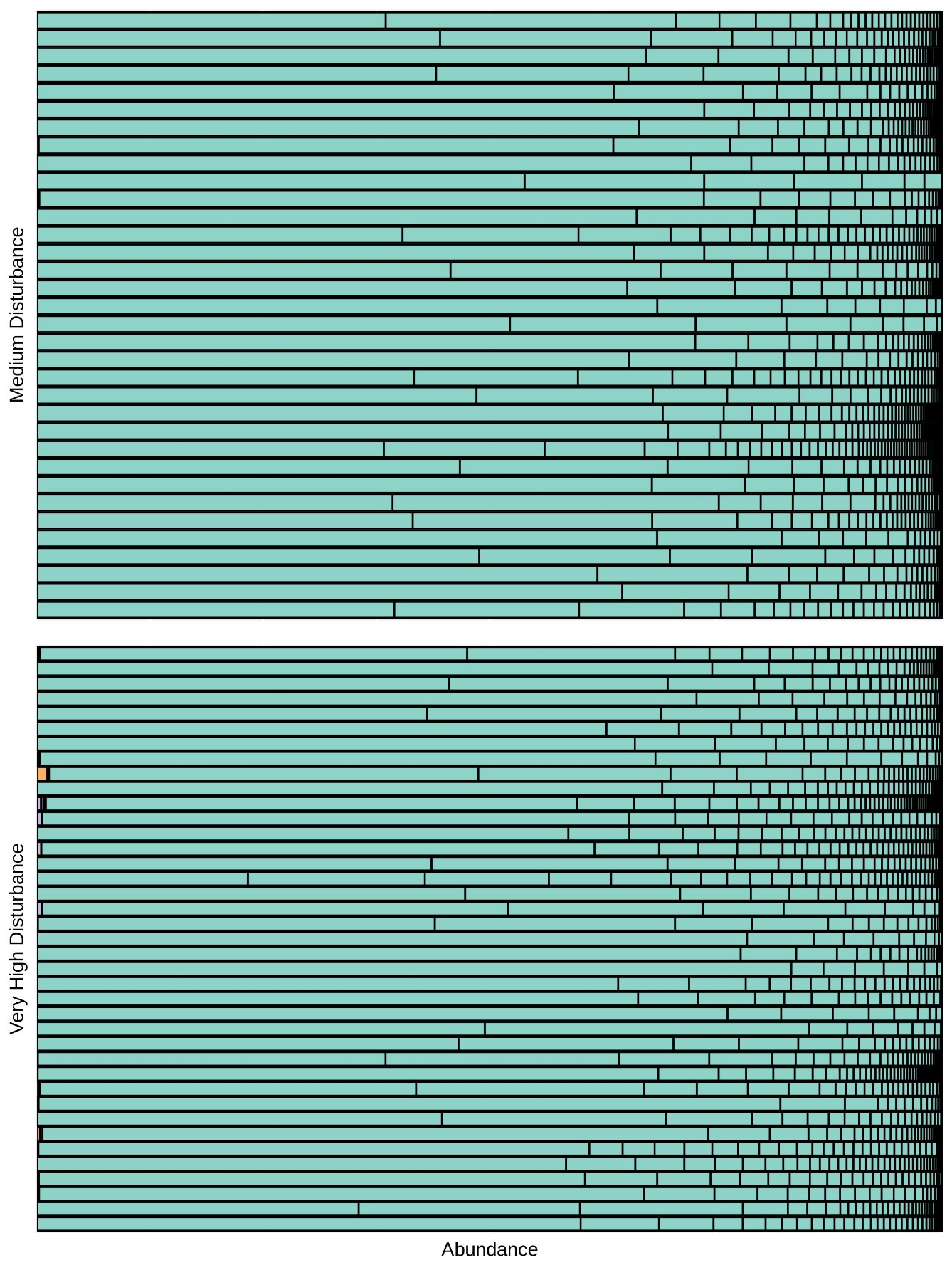


**Supplementary Figure S4. Interpolation-Extrapolation plots for coral a** by disturbance level; **b** by site; **c** by field season


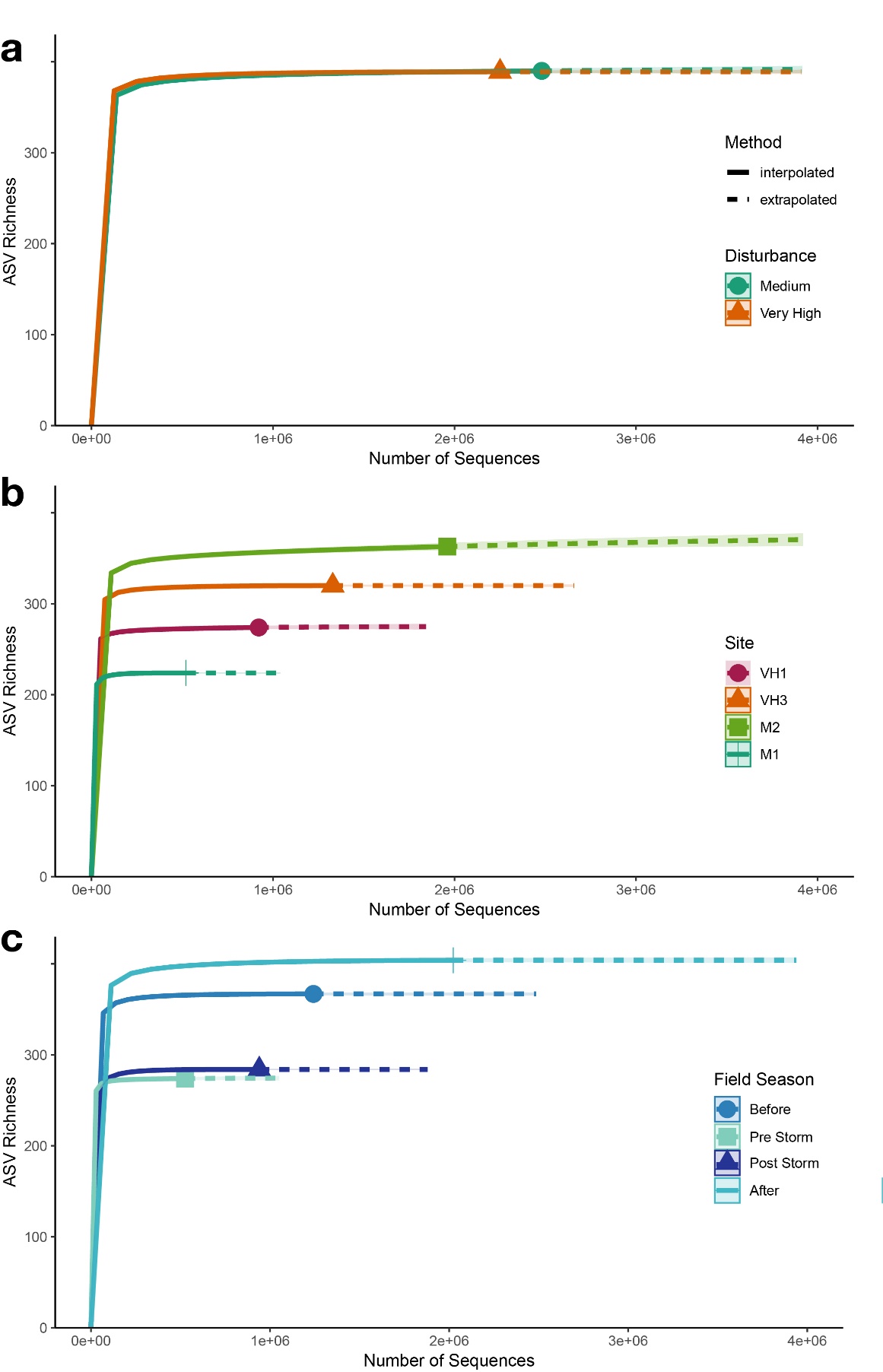


**Supplementary Figure S5. Interpolation-Extrapolation plots for sediment a** by disturbance level; **b** by site; **c** by field season


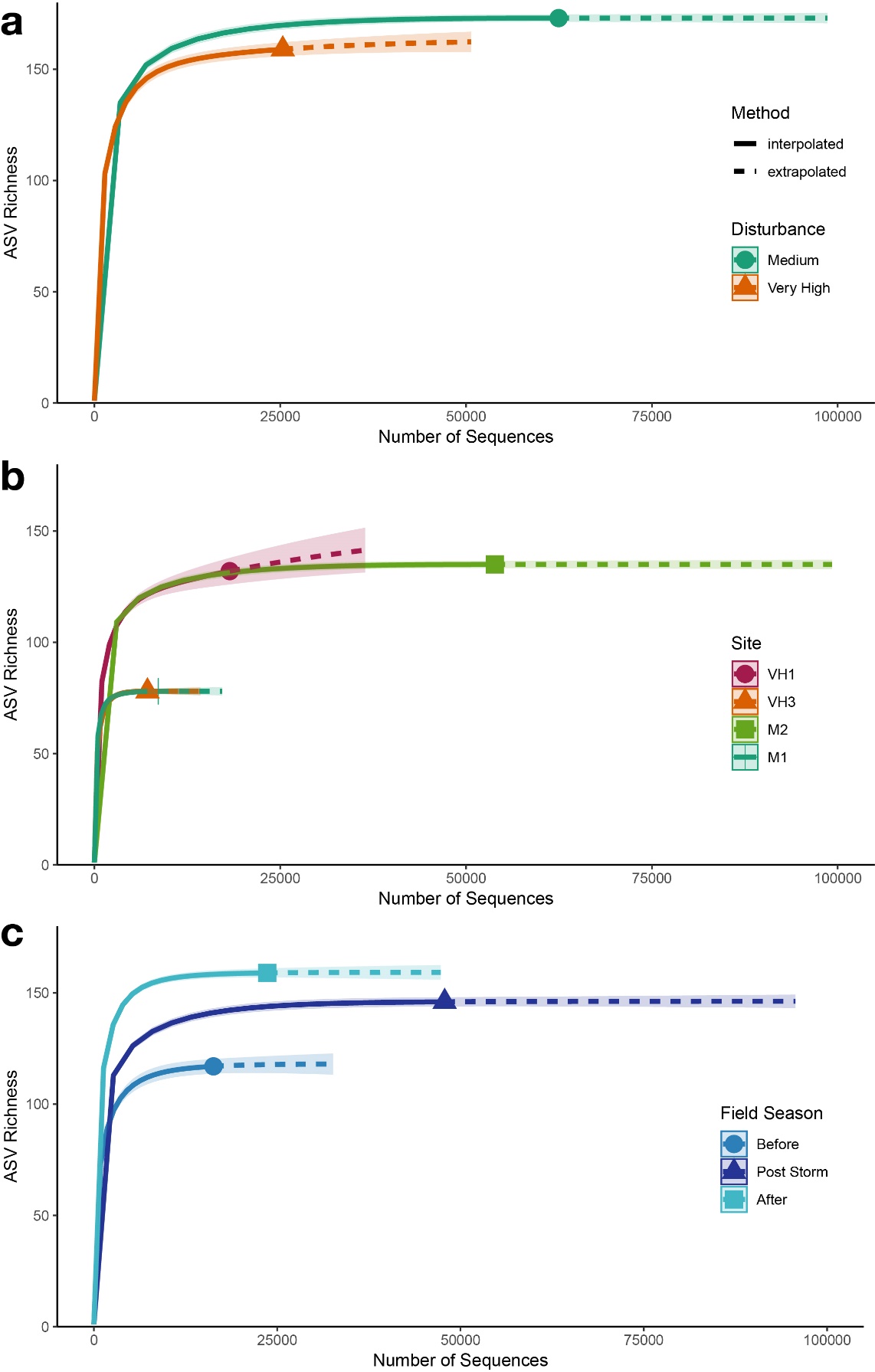


**Supplementary Figure S6. Interpolation-Extrapolation plots for water** **a** by disturbance level; **b** by site; **c** by field season


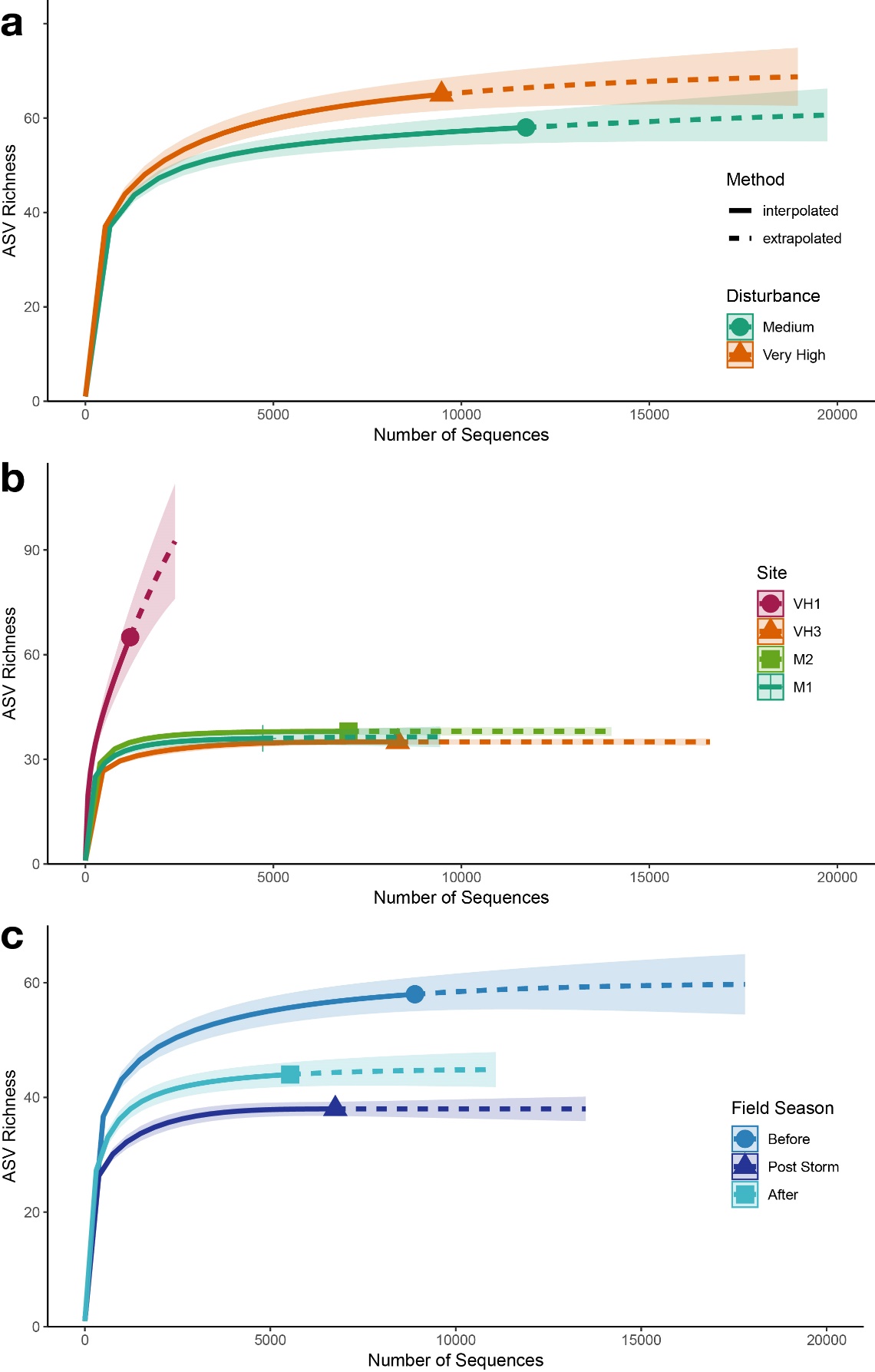


**Supplementary Table S1. Sample size of water, sediment, and corals by site and time point** (August 2014, Before; January 2015, Pre-Storm; January 2015, Post-Storm; May 2015, After). Number of samples reported in “All Coral” is the sum of all samples in *Pocillopora grandis, Montipora aequituberculata,* and *Porites lobata*.

| Site Name | Site  ID | Field Season | Water | Sediment | All Coral | *Pocillopora grandis* | *Montipora aequituberculata* | *Porites lobata* | TOTAL |
| --- | --- | --- | --- | --- | --- | --- | --- | --- | --- |
| M1 | 8 | Before | 4 | 6 | 6 | 2 | 3 | 1 | 16 |
|  |  | Pre-Storm | - | - | - | - | - | - | 0 |
|  |  | Post-Storm | 4 | 5 | 6 | 2 | 3 | 1 | 10 |
|  |  | After | 2 | 5 | 12 | 3 | 6 | 3 | 19 |
|  |  | *Total this Site* | *10* | *16* | *24* | *7* | *12* | *5* |  |
| VH1 | 27 | Before | 4 | 5 | 13 | 3 | 5 | 5 | 18 |
|  |  | Pre-Storm | - | - | - | - | - | - | 0 |
|  |  | Post-Storm | 4 | 6 | 14 | 4 | 5 | 5 | 19 |
|  |  | After | 4 | 6 | 16 | 4 | 7 | 5 | 24 |
|  |  | *Total this Site* | *12* | *17* | *43* | *11* | *17* | *15* |  |
| VH3 | 30 | Before | 5 | 6 | 18 | 5 | 6 | 7 | 29 |
|  |  | Pre-Storm | - | - | - | - | - | - | 0 |
|  |  | Post-Storm | 4 | 6 | 18 | 5 | 6 | 7 | 26 |
|  |  | After | 4 | 5 | 29 | 10 | 9 | 10 | 37 |
|  |  | *Total this Site* | *13* | *17* | *65* | *20* | *21* | *24* |  |
| M2 | 35 | Before | 7 | 6 | 22 | 7 | 7 | 8 | 34 |
|  |  | Pre-Storm | - | - | 16 | 6 | 6 | 4 | 16 |
|  |  | Post-Storm | 4 | 5 | 21 | 8 | 6 | 7 | 30 |
|  |  | After | 4 | 6 | 30 | 11 | 9 | 10 | 40 |
|  |  | *Total this Site* | *15* | *17* | *89* | *32* | *28* | *29* |  |
| **TOTAL** | | | 50 | 67 | 221 | 70 | 73 | 78 | 338 |

**Supplementary Table 2. Calculation of human disturbance categories on Kiritimati.** Intersect_Pop_2km includes the number of people residing within 2 km of each site. Calculated based on Kiribati Population Census report. Fishing Buffer is a kernel density function of fishing intensity with ten discrete levels (from Watson et al. 2016). Intersect_Pop+Fishing is the sum of the previous two columns. Based on these calculations, sites are categorized into five distinct human disturbance categories. Site Name is included for cross-comparisons with other related Kiritimati publications. Note: Sites are part of a larger ecological monitoring program that includes sites with very high, high, medium, low, and very low levels of disturbance.

| **Site Name** | **Intersect_Pop_2km** | **Fishing_Buffer** | **Intersect_Pop+Fishing** | **Disturbance Category** |
| --- | --- | --- | --- | --- |
| VH1 | 4042 | 3234 | 7276 | Very High |
| VH3 | 3065 | 2021 | 5086 | Very High |
| M1 | 0 | 1213 | 1213 | Medium |
| M2 | 0 | 1213 | 1213 | Medium |

**Supplementary Table 3. Environmental parameters on Kiritimati.** *In situ* values are mean ± s.d. for field seasons 2014 and 2015b. Chronic local human disturbance impacts both visibility and coral cover. Visibility was assessed by the same scientific diver pair during each dive. Coral cover was assessed using benthic photographs analyzed in CoralNet ^1^. Temperature, salinity, pH, and DO saturation were collected using a YSI ProPlus handheld sensor that was calibrated daily. Wave intensity and net primary production (NPP) were extracted from satellite data ^2^. Wave energy and NPP Mean represent the general means of each value, and NPP Max represents the mean annual maximum.

| **Disturbance** | **Visibility (m)** | **Coral Cover (%)** |
| --- | --- | --- |
| **Medium** | 16 ± 5 | 41.4 ± 9 |
| **Very high** | 13 ± 2 | 17.2 ± 22 |

| **Disturbance** | **Temperature (°C)** | **Salinity (ppt)** | **pH** | **DO Saturation (%)** | **Wave Energy**  **(kW m^-1^)** |
| --- | --- | --- | --- | --- | --- |
| **Medium** | 29.1 ± 0.05 | 35.1 ± 0.02 | 8.02 ± 0.04 | 98 ± 7 | 25 ± 0 |
| **Very high** | 29.1 ± 0.16 | 35.7 ± 0.84 | 8.05 ± 0.03 | 91 ± 3 | 14 ± 13 |

| **Disturbance** | **NPP Mean**  **(mg C m^-2^ day^-1^)** | **NPP Max**  **(mg C m^-2^ day^-1^)** |
| --- | --- | --- |
| **Medium** | 965 ± 0 | 1078 ± 0 |
| **Very high** | 895 ± 4 | 1104 ± 8 |

**Supplementary Table 4. Volumes of water filtered for each sample.**

| **Sample ID** | **Field Season** | **Site Name** | **Volume (L)** | **Sample Date** |
| --- | --- | --- | --- | --- |
| KI14WSYM004 | 2014 | M1 | 6.0 | 23-Aug-2014 |
| KI14WSYM003 | 2014 | M1 | 8.0 | 23-Aug-2014 |
| KI14WSYM031 | 2014 | M1 | 8.0 | 02-Sep-2014 |
| KI14WSYM021 | 2014 | M1 | 7.2 | 29-Aug-2014 |
| KI14WSYM011 | 2014 | VH1 | 6.5 | 26-Aug-2014 |
| KI14WSYM012 | 2014 | VH1 | 6.3 | 26-Aug-2014 |
| KI14WSYM013 | 2014 | VH1 | 7.1 | 27-Aug-2014 |
| KI14WSYM014 | 2014 | VH1 | 6.1 | 27-Aug-2014 |
| KI14WSYM015 | 2014 | VH3 | 5.5 | 27-Aug-2014 |
| KI14WSYM016 | 2014 | VH3 | 6.5 | 27-Aug-2014 |
| KI14WSYM032 | 2014 | VH3 | 7.6 | 03-Sep-2014 |
| KI14WSYM008 | 2014 | VH3 | 7.7 | 25-Aug-2014 |
| KI14WSYM017 | 2014 | VH3 | 5.7 | 27-Aug-2014 |
| KI14WSYM001 | 2014 | M2 | 7.0 | 21-Aug-2014 |
| KI14WSYM005 | 2014 | M2 | 6.5 | 24-Aug-2014 |
| KI14WSYM028 | 2014 | M2 | 8.6 | 02-Sep-2014 |
| KI14WSYM029 | 2014 | M2 | 8.4 | 02-Sep-2014 |
| KI14WSYM030 | 2014 | M2 | 4.7 | 02-Sep-2014 |
| KI14WSYM006 | 2014 | M2 | 6.2 | 24-Aug-2014 |
| KI14WSYM007 | 2014 | M2 | 7.4 | 24-Aug-2014 |
| KI15aWSYM005 | 2015a | M1 | 8.0 | 1-Feb-2015 |
| KI15aWSYM006 | 2015a | M1 | 8.0 | 1-Feb-2015 |
| KI15aWSYM007 | 2015a | M1 | 9.0 | 1-Feb-2015 |
| KI15aWSYM008 | 2015a | M1 | 10.2 | 1-Feb-2015 |
| KI15aWSYM013 | 2015a | VH1 | 9.0 | 2-Feb-2015 |
| KI15aWSYM014 | 2015a | VH1 | 8.4 | 2-Feb-2015 |
| KI15aWSYM015 | 2015a | VH1 | 8.2 | 2-Feb-2015 |
| KI15aWSYM016 | 2015a | VH1 | 8.8 | 2-Feb-2015 |
| KI15aWSYM017 | 2015a | VH3 | 6.0 | 2-Feb-2015 |
| KI15aWSYM018 | 2015a | VH3 | 7.0 | 2-Feb-2015 |
| KI15aWSYM019 | 2015a | VH3 | 7.6 | 2-Feb-2015 |
| KI15aWSYM020 | 2015a | VH3 | 7.8 | 2-Feb-2015 |
| KI15aWSYM009 | 2015a | M2 | 8.4 | 1-Feb-2015 |
| KI15aWSYM010 | 2015a | M2 | 8.0 | 1-Feb-2015 |
| KI15aWSYM011 | 2015a | M2 | 9.0 | 1-Feb-2015 |
| KI15aWSYM012 | 2015a | M2 | 7.0 | 1-Feb-2015 |
| KI15bWSYM023 | 2015b | M1 | 6.5 | 4-May-2015 |
| KI15bWSYM024 | 2015b | M1 | 7.5 | 4-May-2015 |
| KI15bWSYM017 | 2015b | VH1 | 3.7 | 3-May-2015 |
| KI15bWSYM018 | 2015b | VH1 | 6.2 | 3-May-2015 |
| KI15bWSYM019 | 2015b | VH1 | 7.1 | 3-May-2015 |
| KI15bWSYM020 | 2015b | VH1 | 4.8 | 3-May-2015 |
| KI15bWSYM005 | 2015b | VH3 | 3.9 | 1-May-2015 |
| KI15bWSYM006 | 2015b | VH3 | 4.4 | 1-May-2015 |
| KI15bWSYM007 | 2015b | VH3 | 4.8 | 1-May-2015 |
| KI15bWSYM008 | 2015b | VH3 | 10.5 | 1-May-2015 |
| KI15bWSYM001 | 2015b | M2 | 3.0 | 30-Apr-2015 |
| KI15bWSYM002 | 2015b | M2 | 6.7 | 30-Apr-2015 |
| KI15bWSYM003 | 2015b | M2 | 8.1 | 30-Apr-2015 |
| KI15bWSYM004 | 2015b | M2 | 6.2 | 30-Apr-2015 |

**Supplementary Table 5. Volume of sediment filtered for each sample.**

| **Sample ID** | **Field Season** | **Site Name** | **Sediment (mL)** | **Sample Date** |
| --- | --- | --- | --- | --- |
| KI14SSYM003 | 2014 | M1 | 7 | 23-Aug-2014 |
| KI14SSYM004 | 2014 | M1 | 6.2 | 23-Aug-2014 |
| KI14SSYM005 | 2014 | M1 | 3 | 23-Aug-2014 |
| KI14SSYM006 | 2014 | M1 | 3.5 | 23-Aug-2014 |
| KI14SSYM007 | 2014 | M1 | 3 | 23-Aug-2014 |
| KI14SSYM008 | 2014 | M1 | 2 | 23-Aug-2014 |
| KI14SSYM058 | 2014 | VH1 | 3 | 07-Sep-2014 |
| KI14SSYM059 | 2014 | VH1 | 3 | 07-Sep-2014 |
| KI14SSYM060 | 2014 | VH1 | 3.5 | 07-Sep-2014 |
| KI14SSYM061 | 2014 | VH1 | 3 | 07-Sep-2014 |
| KI14SSYM063 | 2014 | VH1 | 3 | 07-Sep-2014 |
| KI14SSYM015 | 2014 | VH3 | 3.8 | 25-Aug-2014 |
| KI14SSYM016 | 2014 | VH3 | 3 | 25-Aug-2014 |
| KI14SSYM017 | 2014 | VH3 | 3 | 25-Aug-2014 |
| KI14SSYM018 | 2014 | VH3 | 3 | 25-Aug-2014 |
| KI14SSYM019 | 2014 | VH3 | 3 | 25-Aug-2014 |
| KI14SSYM020 | 2014 | VH3 | 3 | 25-Aug-2014 |
| KI14SSYM010 | 2014 | M2 | 3 | 24-Aug-2014 |
| KI14SSYM011 | 2014 | M2 | 3 | 24-Aug -2014 |
| KI14SSYM012 | 2014 | M2 | 3 | 24-Aug -2014 |
| KI14SSYM013 | 2014 | M2 | 3 | 24-Aug -2014 |
| KI14SSYM014 | 2014 | M2 | 3 | 24-Aug-2014 |
| KI14SSYM050 | 2014 | M2 | 3 | 02-Sep-2014 |
| KI15aSSYM025 | 2015a | M1 | 3 | 2-Feb-2015 |
| KI15aSSYM026 | 2015a | M1 | 3 | 2-Feb-2015 |
| KI15aSSYM027 | 2015a | M1 | 3 | 2-Feb-2015 |
| KI15aSSYM029 | 2015a | M1 | 3 | 2-Feb-2015 |
| KI15aSSYM030 | 2015a | M1 | 3 | 2-Feb-2015 |
| KI15aSSYM019 | 2015a | VH1 | 3 | 2-Feb-2015 |
| KI15aSSYM020 | 2015a | VH1 | 3 | 2-Feb-2015 |
| KI15aSSYM021 | 2015a | VH1 | 3 | 2-Feb-2015 |
| KI15aSSYM022 | 2015a | VH1 | 3 | 2-Feb-2015 |
| KI15aSSYM023 | 2015a | VH1 | 3 | 2-Feb-2015 |
| KI15aSSYM024 | 2015a | VH1 | 3 | 2-Feb-2015 |
| KI15aSSYM013 | 2015a | VH3 | 3 | 2-Feb-2015 |
| KI15aSSYM014 | 2015a | VH3 | 3 | 2-Feb-2015 |
| KI15aSSYM015 | 2015a | VH3 | 3 | 2-Feb-2015 |
| KI15aSSYM016 | 2015a | VH3 | 3 | 2-Feb-2015 |
| KI15aSSYM017 | 2015a | VH3 | 3 | 2-Feb-2015 |
| KI15aSSYM018 | 2015a | VH3 | 3 | 2-Feb-2015 |
| KI15aSSYM009 | 2015a | M2 | 3 | 1-Feb-2015 |
| KI15aSSYM010 | 2015a | M2 | 3 | 1-Feb-2015 |
| KI15aSSYM011 | 2015a | M2 | 3 | 1-Feb-2015 |
| KI15aSSYM012 | 2015a | M2 | 3 | 1-Feb-2015 |
| KI15bSSYM019 | 2015b | M1 | 3 | 4-May-2015 |
| KI15bSSYM024 | 2015b | M1 | 3 | 4-May-2015 |
| KI15bSSYM021 | 2015b | M1 | 3 | 4-May-2015 |
| KI15bSSYM023 | 2015b | M1 | 3 | 4-May-2015 |
| KI15bSSYM022 | 2015b | M1 | 3 | 4-May-2015 |
| KI15bSSYM014 | 2015b | VH1 | 3 | 3-May-2015 |
| KI15bSSYM013 | 2015b | VH1 | 3 | 3-May-2015 |
| KI15bSSYM017 | 2015b | VH1 | 3 | 3-May-2015 |
| KI15bSSYM016 | 2015b | VH1 | 3 | 3-May-2015 |
| KI15bSSYM018 | 2015b | VH1 | 3 | 3-May-2015 |
| KI15bSSYM015 | 2015b | VH1 | 3 | 3-May-2015 |
| KI15bSSYM007 | 2015b | VH3 | 3 | 1-May-2015 |
| KI15bSSYM008 | 2015b | VH3 | 3 | 1-May-2015 |
| KI15bSSYM009 | 2015b | VH3 | 3 | 1-May-2015 |
| KI15bSSYM010 | 2015b | VH3 | 3 | 1-May-2015 |
| KI15bSSYM012 | 2015b | VH3 | 3 | 1-May-2015 |
| KI15bSSYM004 | 2015b | M2 | 3 | 30-Apr-2015 |
| KI15bSSYM001 | 2015b | M2 | 3 | 30-Apr-2015 |
| KI15bSSYM006 | 2015b | M2 | 3 | 30-Apr-2015 |
| KI15bSSYM002 | 2015b | M2 | 3 | 30-Apr-2015 |
| KI15bSSYM005 | 2015b | M2 | 3 | 30-Apr-2015 |
| KI15bSSYM003 | 2015b | M2 | 3 | 30-Apr-2015 |

**Supplementary Table 6. Sample sizes from bootstrapped sensitivity analysis.** This table includes only reef habitat compartments that demonstrated significant results for both PERMANOVA (adonis) and PERMDISP (betadisper). Columns “Medium”, “Very High”, “2014”, “2015a”, and “2015b” show original sample size included in the main analysis. Columns “Subsampled to” show the level that samples were subsampled to within each factor level.

|  | **Medium** | **Very High** | **Subsampled to** | **2014** | **2015a** | **2015b** | **Subsampled to** |
| --- | --- | --- | --- | --- | --- | --- | --- |
| **Water** | 25 | 25 | NA |  |  |  |  |
| **Sediment** | 33 | 34 | 33 | 23 | 22 | 22 | 22 |
| ***P. eydouxi*** | 39 | 31 | 31 |  |  |  |  |
| **All samples** | 171 | 167 | 167 |  |  |  |  |

Supplementary References

1. Beijbom, O. *et al.* Towards automated annotation of benthic survey images: Variability of human experts and operational modes of automation. *PLoS ONE* **10**, e0130312 (2015).

2. Yeager, L. A., Marchand, P., Gill, D. A., Baum, J. K. & McPherson, J. M. Marine Socio-Environmental Covariates: queryable global layers of environmental and anthropogenic variables for marine ecosystem studies. *Ecology* **98**, 1976 (2017).
